# La fièvre récurrente à tiques ouest africaine, une maladie négligée mais d’importance majeure en santé publique au Sénégal

**DOI:** 10.48327/mtsi.v5i4.2025.573

**Published:** 2025-09-24

**Authors:** El Hadji Ibrahima NDIAYE, Georges DIATTA, Cheikh SOKHNA, Philippe PAROLA

**Affiliations:** 1 Aix Marseille Univ, RITMES, Marseille, France; 2 IHU Méditerranée Infection, Marseille, France; 3 Campus IRD-UCAD Hann, MINES, IRD 279, Dakar, Sénégal

**Keywords:** *Ornithodoros*, *Borrelia crocidurae*, Fièvre récurrente à tiques, Sénégal, Afrique de l’Ouest, *Ornithodoros*, *Borrelia crocidurae*, Tick-borne relapsing fever, Senegal, West Africa

## Abstract

**Introduction:**

La fièvre récurrente à tiques (FRT), est une zoonose causée par diverses espèces de *Borrelia* transmises aux humains à travers le monde, par la piqûre des tiques molles du genre *Ornithodoros.* Au Sénégal, comme dans les autres régions endémiques d’Afrique de l’Ouest, les cas de FRT, causés par *Borrelia cro-cidurae* et transmis par les tiques *Ornithodoros sonrai,* sont sous-diagnostiqués. La maladie reste peu connue des médecins, des infirmiers et des populations. Dans certaines régions, la FRT est devant le paludisme dans les causes de fièvre à transmission vectorielle.

**Matériel et méthode:**

Cette revue narrative et systématique rapporte des données entomologiques, mammalogiques, épidémiologiques et les aspects cliniques connus de la maladie au Sénégal. Les petits mammifères sauvages, principaux hôtes réservoirs de *B. crocidurae* ont été répertoriés et cartographiés, ainsi que la répartition connue des tiques vectrices. Les méthodes de diagnostic classique comme la visualisation des spirochètes sur une goutte épaisse de sang et/ou un frottis sanguin, mais aussi les méthodes modernes de biologie moléculaire sont décrites, ainsi que le traitement antibiotique.

**Conclusion:**

La stratégie de prévention et de lutte contre la FRT au Sénégal repose sur l’amélioration des conditions d’habitat traditionnel, le remblaiement des terriers et la capture des petits mammifères dans les habitations humaines. Ces méthodes doivent être mieux connues et vulgarisées à travers des campagnes de sensibilisation dans les régions endémiques par les autorités sanitaires.

## Abstract (en)

**Introduction:**

Tick-borne relapsing fever (TBRF) is a zoonotic disease caused by various species of *Borrelia* that are transmitted to humans worldwide through the bite of soft ticks belonging to the genus *Ornithodoros.* In Senegal, as in other endemic regions of West Africa, cases of TBRF caused by *B. crocidurae* and transmitted by *Ornithodoros sonrai* ticks are underdiagnosed. The disease is not well known among doctors, nurses, or the general population. In some regions, TBRF is the leading cause of vector-borne fever, surpassing malaria.

**Materials and methods:**

This narrative and systematic review reports on the entomological, mammalogical, epidemiological, and clinical aspects of TBRF in Senegal. Small wild mammals, the main reservoir hosts of *B. crocidurae,* have been identified and mapped, along with the known distribution of the tick vectors. Conventional diagnostic methods, such as visualization of spirochetes on thick blood smears and/or blood smears, as well as modern molecular biology methods and antibiotic treatment, are described.

**Conclusion:**

The strategy for preventing and controlling TBRF in Senegal involves improving living conditions, filling in burrows, and trapping small mammals in human dwellings. Health authorities in endemic regions need to better disseminate these methods through awareness campaigns.

## Introduction

Les *Borrelia* sont des bactéries spirochètes responsables des borrélioses, qui sont des zoonoses largement répandues dans le monde [27,29,118,119]. On distingue trois groupes principaux de maladies causées par des bactéries du genre *Borrelia* [11,96]. Le premier groupe correspond aux *Borrelia* responsables de la maladie de Lyme, également appelée borréliose de Lyme. Ce groupe comprend environ 20 espèces décrites à travers le monde. Parmi celles-ci, les plus fréquemment impliquées dans les pathologies humaines sont *Borrelia burgdorferi sensu stricto, Borrelia garinii* et *Borrelia afzelii*. Ces espèces sont transmises par des tiques dures appartenant au genre *Ixodes* [11,96]. Le deuxième groupe rassemble les *Borrelia* responsables des fièvres récurrentes transmises principalement par les tiques molles [68,119]. Enfin, le troisième groupe correspond à la fièvre récurrente à poux, causée par *Borrelia recurrentis* transmise par les poux du corps *(Pediculus humanus humanus)* et peut-être ceux de la tête *(P. h. capitis)* [15,17,66,100].

Les formes cliniques de la FRT peuvent être bénignes ou sévères [29,68]. Plus de 14 espèces de *Borrelia* responsables de fièvres récurrentes à tiques sont connues à travers le monde. En Afrique orientale et australe, la FRT causée par *Borrelia duttonii* est bien connue et associée à une létalité significative. En Tanzanie la FRT fait partie des 10 premières causes de mortalité chez les enfants de moins de 5 ans, avec un taux de létalité périnatale de 436/1 000 dans les régions d’endémie [28,73,79].

Elle est transmise par des tiques molles appartenant au groupe *Ornithodoros moubata*, (O. *moubata* et *O. porcinus)* [29,84]. En Afrique de l’Ouest et en Afrique du Nord, *Borrelia crocidurae, Borrelia hispanica* et *Borrelia merionesi* sont les agents de FRT. Ces agents pathogènes sont principalement transmis par plusieurs espèces de tiques molles: *Ornithodoros sonrai, O. marocanus, O. merionesi* et *O. costalis* [117]. *Borrelia hispanica* se distribue dans les zones côtières de l’Afrique du Nord [29,117]. Enfin, *B. crocidurae* est plus répandue en Afrique de l’Ouest et sa distribution couvre également l’Afrique du Nord [29,117]. Les petits mammifères sauvages représentent les principaux réservoirs des *Borrelia* de FRT [38,117,118] à l’exception de *B. duttonii* dont les humains sont le réservoir principal [28].

Au Sénégal, *B. crocidurae* est le seul agent connu de FRT. Elle est transmise par des tiques *O. (Alectorobius) sonrai* qui vivent dans les terriers des petits mammifères sauvages et péri-domestiques [87,117,118]. Dans des zones rurales endémiques du Sénégal, la FRT représentait 11% des motifs de consultations pour syndrome fébrile de 1990 à 2003, avec une prévalence élevée variant de 13% en 2009 à 12% en 2016 [1,88,95,113,121]. *B. crocidurae* a été détectée pour la première fois au Sénégal en 1917 dans le sang d’une musaraigne [69]. Le rôle de la tique *O. sonrai* comme vecteur de la FRT, autrefois appelée fièvre récurrente dakaroise ou spirochétose dakaroise, a été suspecté dans les années 1930 [77]. Un regain d’intérêt pour la maladie et son épidémiologie a eu lieu à partir des années 1990 [36,54,118,121], alors que les cas de FRT étaient souvent cliniquement confondus avec des cas de paludisme [4,36,118]. Avec l’installation de petits laboratoires de diagnostic moléculaire de type *Point-Of-Care* (POC), en milieu rural au Sénégal pour l’identification des causes de fièvres d’origines indéterminées, la maladie a pu être diagnostiquée précocement avec une prise en charge plus rapide [111]. Toutefois, plus de 100 ans après la découverte de cette maladie, la FRT reste mal connue des personnels de santé et des populations du Sénégal [39,88]. Le diagnostic des cas humains reste encore rare en raison de l’absence d’expertise et/ou d’outils de diagnostic simples et accessibles [111]. Les cas humains confirmés sont ainsi le plus souvent rapportés dans des études sur l’identification des causes de fièvre d’origine indéterminée, et la maladie reste encore négligée au Sénégal [1,71,87,88,93,111]. Cette revue narrative et systématique vise à faire le point sur les connaissances actuelles des données cliniques, entomologiques, mammalogiques de la FRT au Sénégal, afin de proposer des méthodes de diagnostic adaptées, modernes et simples, et des programmes d’appropriation des stratégies de lutte dans les régions endémiques.

## Méthodologie de collecte des données

Nous avons effectué une recherche systématique de la littérature dans plusieurs bases de données, notamment Medline, PMC, PubMed, Google, Google Scholar, ainsi que dans des ressources relationnelles et des bibliothèques universitaires pour accéder à des publications anciennes non disponibles en ligne, à partir du 16 octobre 2023. La recherche a été réalisée en utilisant les termes suivants: « tiques » OU « tiques *Ornithodoros* » OU « *Ornithodoros* Sénégal »

OU « *Ornithodoros sonrai* » OU « *Borrelia* » OU « *Borrelia* Sénégal » OU « *Borrelia* récurrente » OU des noms spécifiques d’espèce de *Borrelia*, comme « *Borrelia crocidurae* », etc.; et « fièvre récurrente » OU « fièvre récurrente à tiques » OU « fièvre récurrente à tiques d’Afrique de l’Ouest » OU « fièvre récurrente à tiques au Sénégal ». Ces termes ont été adaptés au format de recherche de chaque base de données.

**Figure 1:**
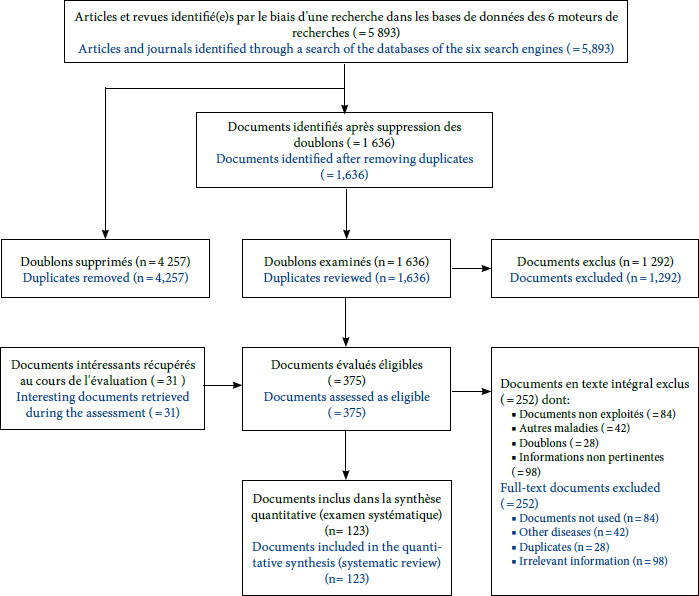
Diagramme de flux du processus de sélection de la documentation pertinente dans la revue systématique

Après avoir supprimé les doublons par Zotero (version 6. 0. 28) et par un tri manuel, nous avons présélectionné les publications en fonction de leur titre et du résumé. Les articles qui ne traitaient pas de la FRT ou qui n’étaient pas en lien avec les objectifs de notre étude (épidémiologie, mam-malogie, entomologie, transmission, vecteurs, réservoirs, portage d’agents infectieux, clinique, diagnostic et traitement de la FRT en Afrique de l’Ouest) ont été exclus. Nous avons ensuite réalisé une revue complète des textes des 375 publications restantes. Les articles qui ne répondaient pas aux critères d’inclusion (portant sur la FRT en Afrique de l’Ouest, particulièrement au Sénégal) ont été éliminés de l’analyse. Nous avons aussi exclu les publications que nous n’avons pas pu obtenir (Fig. 1).

Lors de la revue des textes, nous avons examiné les listes de référence des articles pour trouver d’autres publications pertinentes qui n’auraient pas été repérées dans la recherche initiale. Avec 123 articles éligibles finalement identifiés, nous avons extrait les informations suivantes: premier auteur, titre, année de publication, type et lieu de l’étude, période de l’étude, localisation de l’acquisition/infection par *Borrelia,* espèce de *Borrelia,* espèce de tique vectrice, pourcentage de tiques ou vecteurs infectés par *Borrelia,* petits mammifères réservoirs infectés par *Borrelia* ou non, méthode de diagnostic (microscopie, sérologie, diagnostic moléculaire, inoculation intra péritonéale à la souris blanche adulte *Mus musculus* variété albinos, souche SWISS), degré de certitude diagnostique, nombre de patients, âge (médiane et intervalle), sexe, symptômes, nombre de rechutes de fièvre, grossesses, complications, traitements utilisés et schémas thérapeutiques, nombre de patients traités ou non traités et moyens/méthodes de luttes contre la FRT.

## Historique de la fièvre récurrente à tiques au Sénégal

L’agent de la FRT a été visualisé et décrit pour la première fois en 1917 dans le sang d’un petit mammifère de l’ordre des Eulipotyphles, une musaraigne initialement identifiée *Crocidura stampflii,* prise au piège dans les égouts de la ville de Dakar [69]. Dans le cadre de ses recherches sur le rôle des animaux comme réservoirs de maladies parasitaires, André Léger a observé un spirochète dont la description différait de celle des autres bactéries connues. Sa particularité résidait dans la formation de chaines composées de 3 à 6 spirochètes disposés bout à bout ou d’amas en écheveau. Chaque organisme visualisé au microscope se présentait sous forme d’un filament enroulé en spirales et terminé par des extrémités effilées, sans pour autant qu’il soit permis de trouver des cils ou des flagelles véritables. Il possédait 4 ou 5 ondulations sur le même plan et mesurait environ 14 à 16 µm de longueur et 2 à 5 pm de largeur [69]. Ainsi, il proposa de nommer « *Spirochaeta crocidurae* » ce spirochète qui sera renommé par la suite *Borrelia crocidurae.*

En 1920, des spirochétoses sanguicoles humaines ont été signalées à Dakar [90], et en 1923, des rongeurs ont été retrouvés également infectés par des spirochètes sanguicoles [70]. Mathis (1926) a démontré, à travers des infections expérimentales réalisées sur des animaux en laboratoire, que deux sujets humains avaient été contaminés par des spirochètes provenant de rats capturés à Dakar. Il a ainsi établi que les spirochètes sanguicoles identifiés chez la musaraigne pouvaient être pathogènes pour les humains. Les symptômes observés chez les sujets infectés étaient identiques à ceux du « typhus récurrent humain » [74]. À cette époque, un foyer endémique de cette maladie sévissait à Dakar dont l’agent était inconnu [75]. Il a ensuite mis en évidence la similitude entre les spirochétoses sanguicoles humaines et celles observées chez les petits mammifères. Il a également souligné le rôle de la musaraigne, ainsi que celui de divers petits rongeurs muridés, en tant que réservoirs de l’agent pathogène responsable du typhus récurrent humain. Il a proposé de désigner cette forme endémique observée à Dakar sous le nom de fièvre récurrente à tiques (FRT) dakaroise [76,77]. Une tique molle du complexe *Ornithodoros erraticus,* collectée dans les terriers des petits mammifères de Dakar, a été désignée comme responsable de la transmission aux humains de la FRT dans les années 30 [46,77].

Ces tiques étaient infectées par un spirochète identique à celui retrouvé chez les sujets atteints de spirochétose dakaroise, ainsi que dans le sang de la musaraigne et de petits rongeurs muridés [76]. En 1949, Boiron a déclaré que l’indice de contamination de la FRT dakaroise par les tiques du complexe *O. erraticus* dépendait de la nature des habitations et de celle de leur sol [13].

À la fin des années 40, l’attention des médecins coloniaux d’Afrique occidentale est attirée par l’infection humaine causée par *Spirochaeta crocidurae,* l’agent de la FRT dakaroise. Les observations cliniques se multiplient, décrivant les différentes formes cliniques et les complications de la maladie [10]. La caractéristique la plus notable est l’aspect de la courbe thermique. Cette anomalie s’accompagne de manifestations hépato-vésiculaires, ictériques, rénales et neurologiques, comme des formes méningées pures, des troubles oculaires et des paralysies périphériques et médullaires [10]. La maladie est alors caractérisée par le syndrome de fièvre à rechutes de Larrey avec une succession périodique d’épisodes fébriles séparés par des intervalles d’apyrexie, d’où le futur nom de FRT donné à la maladie [101]. Bergeret et Raoult recommandaient sur la simple notion de récurrence fébrile d’un patient en zone endémique, de rechercher le spirochète de la FRT dakaroise [9,10]. En même temps, ils établissaient les bases thérapeutiques pour traiter les patients avec certaines molécules comme le novarsénobenzole, sulfarsénol, orsanine, tryparsamide, acétylarsan, diamidine et la pénicilline qui ont donné des résultats satisfaisants [10]. À Dakar, les cas annuels hospitalisés étaient passés de 26 en 1942 à 86 en 1946 [9,76,77]. Ainsi, les connaissances cliniques et épidémiologiques sur la FRT d’Afrique de l’Ouest et du Nord provenaient en grand partie des médecins et des hôpitaux de Dakar [10].

Après une longue période de diminution de l’activité de recherche sur la FRT, voire d’oubli, un regain d’intérêt pour cette maladie s’est manifesté dans les années 1990. De nouvelles études ont débuté avec le suivi des populations à l’aide de la goutte épaisse de sang. Entre 1989 et 1990, des infections à *B. crocidurae* ont été détectées chez 0,9% des enfants de moins de 15 ans consultant au dispensaire de Keur Moussa, dans la région de Thiès [118]. Dans cette région, une prévalence de *B. crocidurae* de 4,2% a été mise en évidence par la goutte épaisse chez des patients fébriles qui y étaient examinés [118]. De juin 1990 à mai 1992 à Dielmo, village dans la région de Fatick, le taux d’incidence annuel moyen de la FRT était de 5,2% chez des malades présentant un accès fébrile [36,120]. Selon les années, le suivi clinique et parasitologique de la population villageoise de Dielmo, réalisé pendant 14 ans, a montré qu’en moyenne 11% de la population développait la maladie, le taux d’incidence annuel de la FRT fluctuant de 4% à 25% [121]. L’identification des infections à *Borrelia* été rendue plus facile avec l’apport des outils moléculaires de type POC, un petit laboratoire de diagnostic basé sur des analyses de biologie moléculaire de type PCR ou qPCR [111]. Ainsi en 2010, une prévalence de la FRT a été évaluée à 13% par les analyses de biologie moléculaire, alors que les examens par la goutte épaisse de sang n’avaient visualisé les *Borrelia* que chez 2% des patients fébriles [95]. Au cours des 30 dernières années, les connaissances sur la FRT au Sénégal se sont encore précisées. Cela concerne plusieurs domaines: la bactériologie, l’épidémiologie, les aspects cliniques, entomologiques et mammalogiques [4,68,83,87,88,117]. La FRT a vraisemblablement représenté la deuxième cause de consultation pour maladies à transmission vectorielle, après le paludisme. Actuellement, et dans un contexte de lutte accrue contre le paludisme, elle constitue également la principale maladie vectorielle dans certaines zones rurales endémiques au Sénégal [27,29,118,120].

En septembre 2022, dans la zone de Niakhar à Fatick, l’incidence mensuelle des cas d’infection par la borréliose à tiques variait entre 11 et 23, avec une prévalence de 12%. Une campagne de sensibilisation de masse a été menée auprès de la population, avec information sur les moyens de lutte. Grâce à cette intervention, l’incidence mensuelle des cas humains a diminué en 2023, passant à seulement 1 à 5 cas par mois, sur une population estimée à plus de 45 000 habitants (Diatta G, données non publiées) [33]. C’est au cours de ces rassemblements d’information et sensibilisation, qui réunissait tous les chefs de village ainsi que les autorités administratives et sanitaires, que l’assemblée constitutive a pris une décision importante. Elle a attribué l’appellation « Sibiru Magaré Ndiock » comme nom vernaculaire en langue sérère de la FRT due à l’infection à *Borrelia* chez les souris, c’est-à-dire les petits rongeurs.

### Borrelia crocidurae

Anciennement connue sous le nom de « *Spirocheata crocidurae* » [69,110], cette bactérie a donc été officiellement rebaptisée *Borrelia crocidurae* par Davis [110]. Les *Borrelia* sont des bactéries spiralées (spirochètes), plus ondulées que spiralées, mesurant 3 à 25 µm de long sur 0,2 à 0,5 pm de large [47]. Elles sont toutes morphologiquement identiques et possèdent un corps grêle et des extrémités effilées (Fig. 2A et 2B). Les *Borrelia* ne possèdent pas de véritables flagelles, mais des endoflagelles, au nombre de 7 à 30, situées dans l’espace péri-plasmique. Ces endoflagelles forment une hélice flexible assurant leur mobilité caractéristique [107]. Ce sont des organismes dont le génome comprend un chromosome linéaire d’une taille allant de 900 000 à 920 000 paires de bases (pb), ainsi que plusieurs plasmides circulaires et linéaires, certaines espèces pouvant en posséder jusqu’à 20 différents [107]. Toutes les *Borrelia* ont le même cycle évolutif au cours de leur vie. Elles sont extra cellulaires et vivent en milieu liquide (plasma, hémolymphe de l’arthropode vecteur) ou dans les espaces intercellulaires des tissus [7,53]. La détection de ces spirochètes dans les tissus peut s’effectuer après imprégnation argentique. Elles réagissent à la coloration de Giemsa qui leur donne une teinte mauve ou bleu-violet [37]. *B. crocidurae* peut être cultivée *in vitro* sur milieu de Kelly modifié [32,95,117].

Le génome de *B. crocidurae* (1 557 560 pb, 27% de GC) a été publié [62]. L’analyse des séquences partielles concaténées des *Borrelia* chez les tiques *Ornithodoros* a contribué à l’étude des relations phylogénétiques et de la répartition géographique de *B. crocidurae* en Afrique de l’Ouest (Fig. 3A et 3B) [117]. Le MALDI-TOF MS, une technologie de spectrométrie de masse devenue une référence pour l’identification rapide, fiable et à faible coût des bactéries, a été utilisée pour l’identification des *Borrelia.* Cette méthode repose sur l’ionisation douce des protéines par une matrice, suivie d’une séparation en fonction du rapport masse/charge dans un analyseur à temps de vol. Elle permet ainsi de générer des profils protéiques spécifiques, propres à chaque espèce, facilitant leur discrimination (Fig. 4A) [16,20]. Cet outil a également permis de distinguer des tiques *O. sonrai* infectées ou non par *B. crocidurae* (Fig. 4B) [50].

**Figure 2A:**
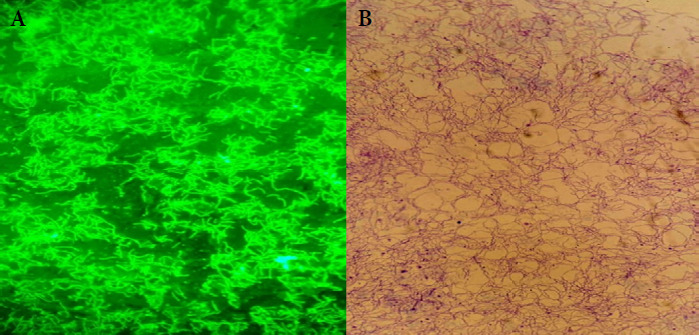
Visualisation de *Borrelia crocidurae* cultivées au laboratoire de l’IHU Méditerranée Infection à Marseille: immunofluorescence indirecte (A); sérum de souris, objectif à filtre UV (x40) et coloration de Gimenez (B); objectif à immersion (x1000)

**Figure 2B:**
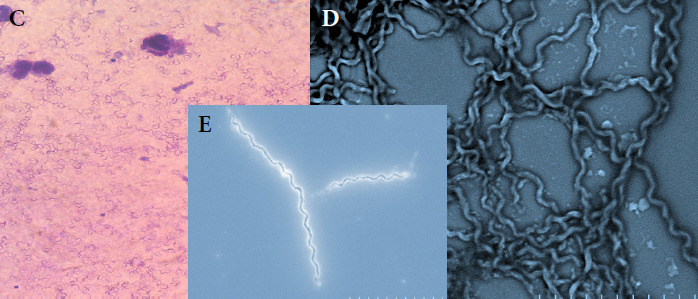
Visualisation de *B. crocidurae* au microscope par coloration Giemsa (C): amplifiés dans le sang de souris blanche, variété albinos, souche SWISS, objectif à immersion (x1000); fixation au glutaraldéhyde (D&E): souches de l’IHU Méditerranée Infection à Marseille, microscope électronique à balayage SU5000 BSE-ALL

**Figure 3A:**
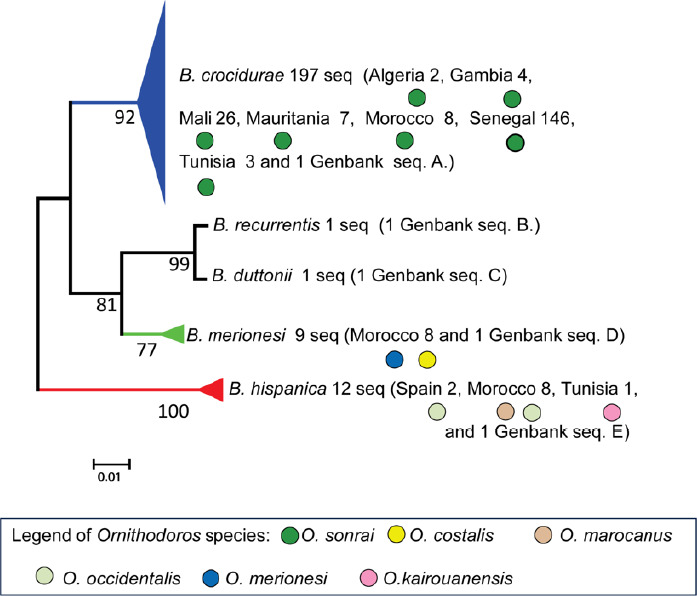
Relations phylogénétiques entre les espèces de *Borrelia* à l’aide de séquences concaténées d’espaceurs intergéniques partiels (IGS, 510 nucléotides) et de séquences concaténées de gènes FlaB partiels (FLA, 269 nucléotides) (PhyML 100 bootstraps, disponible sur http: //mobyle.pasteur.fr/cgi-bin/portal.py). Le triangle coloré permet d’estimer la diversité des espèces de *Borrelia.* Trape *et al.,* 2104, ont inclus dans le clade *B. merionesi* (9 seq.) une *Borrelia* détectée chez un rongeur capturé à El Argoub (Maroc). Le cercle plein coloré correspond à l’espèce de tique déterminée par l’analyse phylogénétique 16S de cette étude. Le phylogramme a été construit en utilisant une méthode de vraisemblance maximale à partir de données de séquences concaténées (220 séquences incluant les séquences de référence GenBank, 779 nucléotides). Les valeurs de bootstrap >70 sont indiquées (échelle, 0,01 substitutions par site). Seq. A.: *B. crocidurae* (GU350723 et NC017808), seq. B.: *B. recurrentis* (DQ000277 et DQ346814), seq. C.: *B. duttonii* (DQ000279 et DQ346833), seq. D.: *B. merionesi* (JX257047 et JX257050), seq. E.: *B. hispanica* (GU350718 et GU357614) et les séquences concaténées ont été utilisées comme références.doi:10.1371/journal.pone.0078473.g004, Trape *et al.,* 2013

**Figure 3B:**
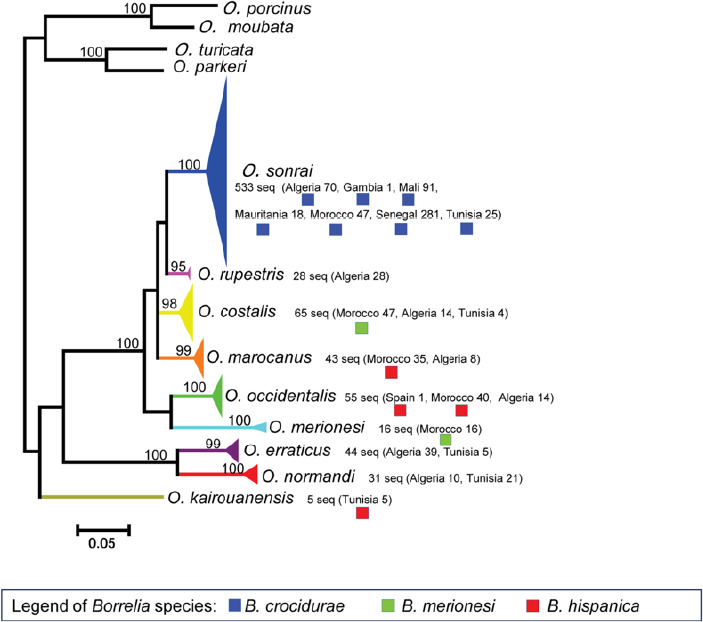
Relations phylogénétiques entre les espèces d*‘Ornithodoros à* l’aide de séquences d’ARNr 16S (820 seq.). Les triangles colorés indiquent la diversité génétique des séquences détectées pour chaque espèce *d’Ornithodoros.* Les points colorés correspondent aux espèces de *Borrelia* détectées dans chaque espèce de tique. Le phylogramme a été construit en utilisant une méthode de vraisemblance maximale à partir de données de séquences partielles 16S (457 nucléotides). Les valeurs Bootstrap > 90 sont indiquées (barre d’échelle, 0,05 substitutions par site). *Ornithodoros moubata* (numéro d’accès GenBank AB073679), *Ornithodoros porcinus* (numéro d’accès GenBank AB105451), *Ornithodoros turicata* (numéro d’accès GenBank L34327) et *Ornithodoros parkeri* (numéro d’accès GenBank EU009925) ont été traités comme des groupes externes. Cinq de ces espèces sont nouvellement décrites: *Ornithodoros occidentalis, Ornithodoros costalis, Ornithodoros rupestris, Ornithodoros. kairouanensis* et *Ornithodoros merionesi* doi:10.1371/journal.pone.0078473.g002,Trape *et al.,* 2013

**Figure 4A:**
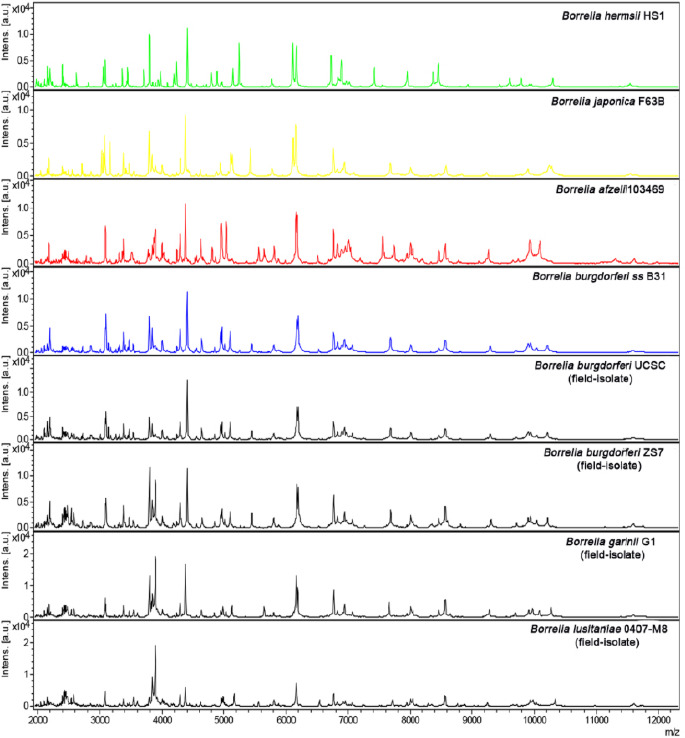
Analyse MALDI-TOF MS des souches de référence de quatre espèces de *Borrelia* et d’isolats de terrain provenant à la fois d’humains et de tiques. Spectres obtenus par analyse MALDI-TOF MS des souches de référence de quatre espèces de *Borrelia (B. hermsii* HS1 en vert, *B. japonica* F63B en jaune, *B. afzelii* 103469 en rouge, *B. burgdorferi* ss B31 en bleu) et d’isolats de terrain d’origine humaine (B. *burgdorferi* ss UCSC et B. garinii G1) et de tiques (B. *burgdorferi* ZS7 et *B. lusitaniae* MT 0407-M8), tous en noir.doi:10.1371/journal.pone.0088895.g001, Calderaro *et al.,* 2014.

**Figure 4B:**
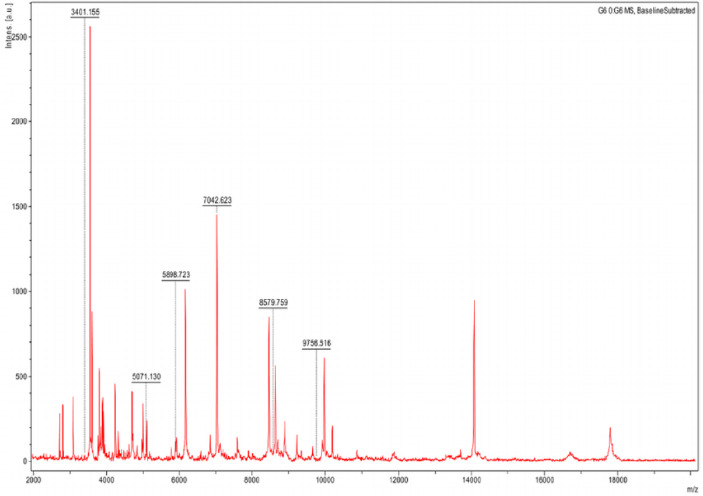
Un modèle à 6 pics pour la détection spécifique de *B. crocidurae* chez les tiques *O. sonrai.* doi:10.1371/journal.pntd.0002984.q002, Fotso Fotso *et al.,* 2014

### *Ornithodoros sonrai* et transmission

**Figure 5:**
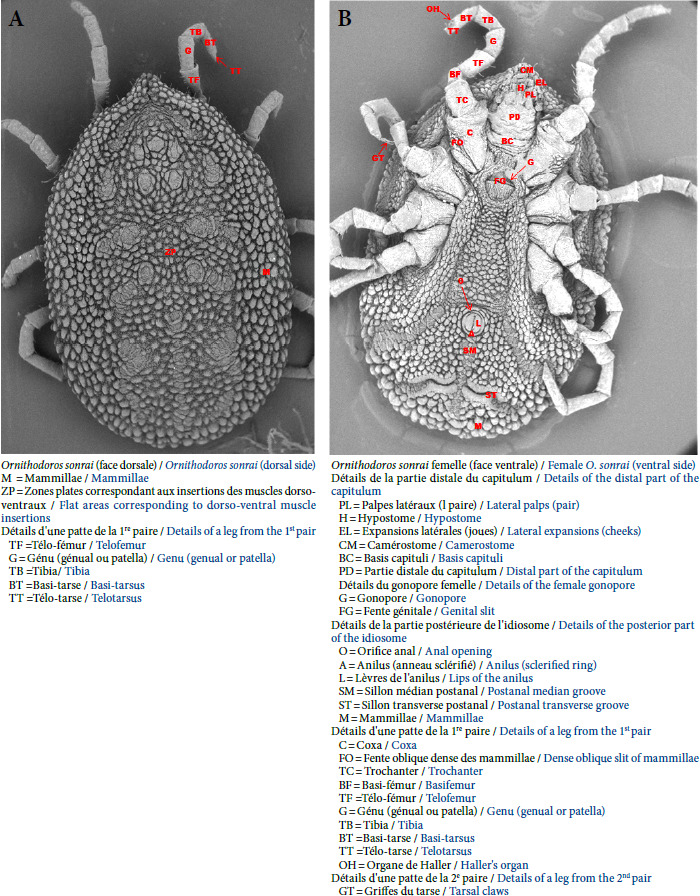
Détails des caractéristiques morphologiques dorso-ventrale d’une tique *O. sonrai* du Sénégal au microscope électronique à balayage

Les tiques du genre *Ornithodoros* sont de la famille des Argasidés, encore appelées tiques molles. Leur corps épais, non segmenté, de forme ovale, est formé de deux régions: l’idiosome et le capitulum situé en position ventrale [36]. Elles n’ont pas d’yeux. Les adultes se différencient du stade nymphal notamment par la position ventrale du capitulum. Au stade larvaire, le capitulum est séparé de l’idiosome et en position terminale [103]. Le capitulum est situé dans une cavité de l’idiosome appelée le camérostome. Ce capitulum porte les organes des sens (palpes) et les pièces buccales (hypostome et chélicères), et le camérostome possède des expansions latérales appelées joues [36]. Le tégument de l’idiosome est pourvu de petits tubercules appelés mammillae [36]. Sur la face dorsale, la cuticule présente des zones plates où les mammillae sont effacées et correspondent aux insertions des muscles dorso-ventraux [36]. L’idiosome porte ventralement l’insertion des pattes et les orifices, génital et anal. Les pattes (quatre paires chez les adultes et les nymphes et trois paires chez les larves) s’insèrent directement sur le tégument par une pièce fixe ou coxa [63,103] (Fig. 5).

La tique *O. sonrai* de petite taille (3 à 5 mm pour les adultes) est classée au sein du complexe *O. erraticus* actuellement composé de 9 espèces génétiquement différentes et morphologiquement proches [106,117,121]. Les tiques *O. sonrai* sont comme les autres ornithodores, des ectoparasites obligatoires. Elles sont endophiles, vivant dans la nature en colonisant les terriers des petits mammifères, ce qui leur procure des conditions de microclimat favorable à leur développement [103,117]. On distingue trois stases (larvaire, nymphal et adulte) qui passent par plusieurs stades de métamorphose parfois appelées « mues » au cours de leur cycle de développement [36,103,119].

**Figure 6:**
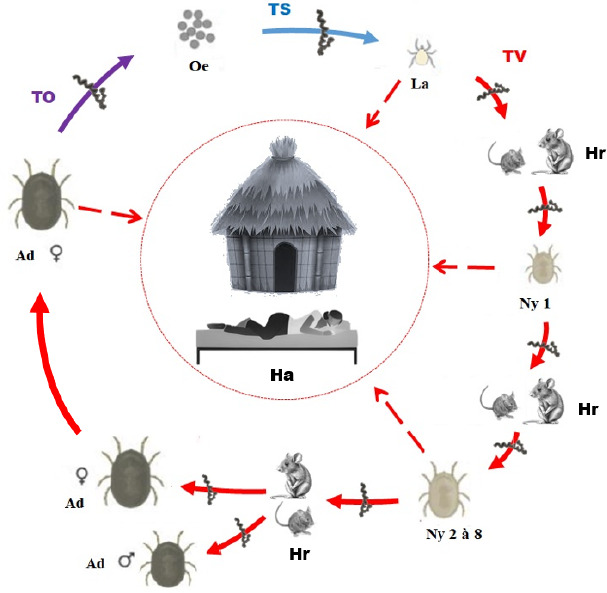
Circulation de *B. crocidurae* dans le cycle parasitaire de la tique *O. sonrai* et transmission accidentelle de la fièvre récurrente à tiques aux humains

Le cycle de développement d’O. *sonrai* comporte deux phases, une parasitaire sur le rongeur-hôte et une libre, au sol (Fig. 6). La phase parasitaire est de courte durée, avec un nombre important de repas sanguins. Les adultes effectuent un repas sanguin avant chaque accouplement [102]. La piqûre des tiques *Ornithodoros* est indolore et se produit généralement la nuit pendant le sommeil dans les habitations traditionnelles au sol en terre battue et fréquentées par des rongeurs. Pendant le repas, le liquide coxal libéré est la source de la transmission des *Borrelia* [101]. Les tiques abandonnent l’hôte après leur repas qui dure environ 10 à 20 minutes pour poursuivre leur cycle de vie au sol [103]. Les femelles *O. sonrai* pondent des œufs au sol dans le terrier au bout de 13 jours maximum. En zone tropicale, la reproduction des tiques *O. sonrai* adultes se déroule toute l’année. La durée de vie des tiques *Ornithodoros* est longue (comprise entre 10 à 20 ans selon les espèces et les conditions de température et d’humidité relative), ce qui explique leur présence dans les terriers abandonnés par les rongeurs [38,103,119].

Les *O. sonrai* peuvent ingérer des *Borrelia* (Fig. 6) durant leur repas de sang à partir d’un vertébré infecté, le plus souvent des petits mammifères qui représentent les principaux réservoirs de bactéries [68]. Les *Borrelia* ingérées se développent dans l’intestin de la tique au contact de la muqueuse intestinale, puis migrent activement vers les glandes salivaires et coxales [51,107]. La transmission de l’infection au vertébré intervient au cours d’un repas sanguin de la tique infectée par l’intermédiaire des sécrétions des glandes salivaires et coxales [21,107]. Le passage de *B. crocidurae* de la tique *O. sonrai* aux principaux hôtes vertébrés réservoirs, et *vice versa,* est nécessaire à la propagation de l’agent pathogène *B. crocidurae* et à l’établissement d’une endémicité [38,88,117].

Il existe aussi chez les tiques une transmission trans-ovarienne et trans-stadiale des *Borrelia.* Au cours de ce processus, les descendants de la femelle *O. sonrai* infectée deviennent eux-mêmes infectants. L’infection se transmet également d’un stade de développement à l’autre, ce qui permet d’assurer le maintien de l’agent pathogène *B. crocidurae* chez les tiques *O. sonrai* (Fig. 6). Les tiques peuvent donc également jouer le rôle de réservoir de bactérie. Les *Borrelia* peuvent se maintenir plusieurs années dans la tique sans perdre leur pouvoir pathogène [68,112]. La longévité des tiques *Ornithodoros* et le maintien de leur potentiel vectoriel ont été démontrés chez la tique *Ornithodoros tholozani* capable de transmettre les *Borrelia* après 11 ans [98]. Cependant, la transmission chez les tiques peut s’accompagner d’une diminution progressive de la charge bactérienne au fil du temps [51,107].

### *Ornithodoros sonrai* et les micro-organismes associés au Sénégal

**Figure 7:**
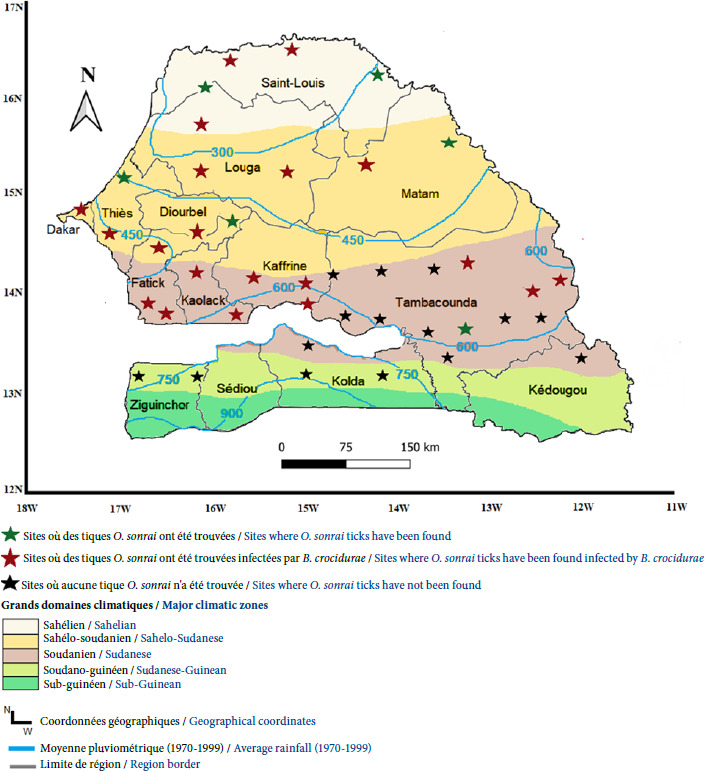
Distribution de *B. crocidurae* détectée chez des tiques *O. sonrai* de 1991 -2022 au Sénégal

Au Sénégal, *O. sonrai* colonise les terriers de petits rongeurs des régions saharienne, sahélienne, et une partie de ceux situés au nord de l’isohyète 750 mm de pluie en région soudano-sahélienne [117]. La limite de distribution sud se situe à la frontière entre le Sénégal et la Gambie et correspond à la position de l’isohyète 750 mm (Fig. 7) [117]. La tique est absente dans les régions où la pluviométrie est supérieure à l’isohyète 750 mm notamment à Ziguinchor, Kolda, Sédhiou, Kédougou et au sud de la région de Tambacounda, correspondant approximativement à la latitude 13°15’N [117]. *Borrelia crocidurae* a été retrouvée chez les tiques *O. sonrai* collectées dans les 2/3 nord du Sénégal [10,37,77,117,120]. Les outils de biologie moléculaire ont permis de préciser la distribution géographique de *B. crocidurae* chez les tiques *O. sonrai* collectées dans les zones endémiques du Sénégal (Fig. 7) [42,88,117]. En plus de son rôle de porteur de l’infection à *B. crocidurae, O. sonrai* peut héberger d’autres micro-organismes comme *Coxiella burnetii,* l’agent de la fièvre Q, ainsi que des bactéries des genres *Bartonella*, *Wolbachia, Ehrlichia,* et *Anaplasma.* Elle héberge également *Occidentia massiliensis,* un membre de la famille des Rickettsiaceae dont la pathogénicité demeure inconnue [80-82,86].

### *Borrelia crocidurae* chez les humains

Le cycle de développement des *Borrelia* chez les humains commence par une multiplication active de la bactérie dans le compartiment vasculaire. Les *Borrelia* présentent un tropisme préférentiel pour l’endothélium vasculaire, le système nerveux central, le foie, la rate et la moelle osseuse dont le mécanisme n’a pas été encore élucidé [19,99,105]. *Borrelia crocidurae* a la capacité de s’agréger aux érythrocytes de l’hôte, ce qui conduit à la formation d’agrégats appelés rosettes érythrocytaires. Ces structures perturbent l’interaction entre les cellules immunitaires et les bactéries, entraînant ainsi un retard de la réponse immunitaire. Par ailleurs, la présence de ces agrégats peut provoquer des micro-embolies, à l’origine de lésions tissulaires [18,109]. En outre, *B. crocidurae* a, en commun avec les *Borrelia* du complexe *B. burgdorferi,* la capacité d’activer les cellules de l’endothélium vasculaire et de favoriser la migration trans-endothéliale des polynucléaires neutrophiles [109]. Ces événements entraînent des lésions vasculaires et des réactions inflammatoires qui représentent, avec la formation des micro-embolies, la base de la physiopathologie de la FRT à *B. crocidurae.*

Ces *Borrelia* sont le plus souvent visibles dans le sang périphérique durant les périodes de fièvre. Elles disparaissent ou sont très rares pendant les périodes d’apyrexie, et la récurrence thermique correspond à une récurrence de bactériémie [5]. Les *Borrelia* sont capables de modifier leur structure antigénique de surface et d’échapper pour un temps au système immunitaire [5]. Les anticorps spécifiques qui apparaissent lors du premier épisode bactériémique permettent la destruction de la quasi-totalité des *Borrelia* présentes dans le sang [89]. Cependant, ces anticorps ne seraient actifs que pour une seule récurrence, et ne peuvent être dirigés contre les antigènes d’une nouvelle protéine de surface [8]. Ce phénomène d’échappement immunitaire est à l’origine des récurrences bactériennes. L’incubation dure sept jours en moyenne, et la maladie débute par un frisson suivi d’une poussée fébrile à 40 °C [47,123]. Les épisodes de fièvre peuvent être accompagnés de céphalées, de douleurs articulaires, musculaires et osseuses, et fréquemment de troubles digestifs tels que des douleurs abdominales, des vomissements et des diarrhées [68,91,97]. On peut également observer une hépato-splénomégalie et une anémie [68,91,97]. Après une période de fièvre de 3 à 4 jours, l’accès fébrile se termine par une chute thermique rapide et sudorale, et une chute tensionnelle [47,68,97]. Ensuite, suit une période d’apyrexie de 2 à 10 jours au cours de laquelle le patient continue de sentir des douleurs musculaires et osseuses [47,68]. Les patients non traités présentent alors jusqu’à neuf récurrences sur plusieurs mois, des épisodes de fièvre espacés de quelques jours [9,47]. Les signes neurologiques apparaissent généralement au cours du deuxième épisode fébrile [19]. Des complications de méningo-encéphalites, d’hépatonéphrites, d’atteintes oculaires et d’avortements spontanés chez les femmes enceintes peuvent survenir à tout moment de l’évolution de la maladie [10,23,49,55,56,68,102]. Cependant, la paralysie faciale, souvent observée comme parmi les principaux signes et symptômes de neuro-borréliose causés par les espèces du complexe *B. burgdorferi sl.,* n’a pas été observée avec *B. crocidurae* [19]. En pratique, le cadre clinique est souvent celui d’une « fièvre d’étiologie indéterminée », bien connu en zone tropicale.

### Le réservoir naturel de *B. crocidurae* au Sénégal

Si la transmission verticale des *Borrelia* de la FRT chez les tiques pérennise le maintien des foyers endémiques de borréliose dans l’environnement [98,108], les petits mammifères sauvages et péri-domestiques constituent des réservoirs [13,22,41,88,114,117,119,120]. Au Sénégal, plus de 30 espèces de petits rongeurs et d’eulipotyphles ont été trouvées naturellement infectées par *B. crocidurae* [38,41,59,117-121], ainsi que par d’autres micro-organismes [30,38,45,59]. Au Sénégal, plus de quatorze espèces de rongeurs, ainsi qu’une musaraigne et un hérisson (eulipotyphle) ont été répertoriées comme porteurs de *B. crocidurae* [37,38,54,119].

**Figure 8:**
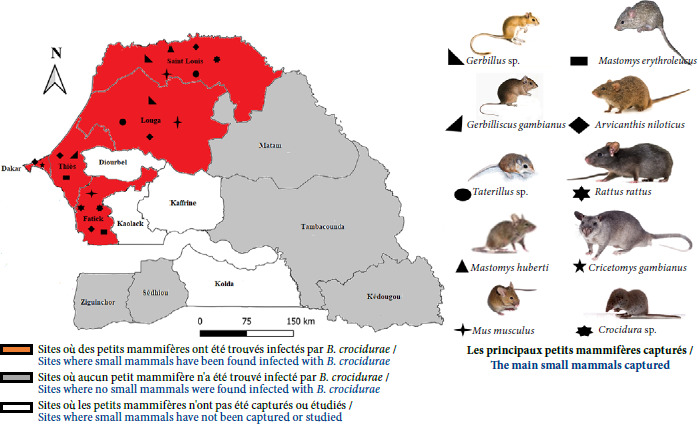
Distribution de *B. crocidurae* détectée chez différentes espèces de micromammifères au Sénégal

On peut notamment citer les rongeurs *Mus musculus domesticus, Mastomys erythroleucus, Mastomys natalensis, Mastomys huberti, Rattus rattus, Rattus norvegicus Arvicanthis niloticus, Cricetomys gambianus, Heliosciurus gambianus, Desmodilliscus braueri, Tatera gambiana (Gerbilliscus gambianus), Taterillus gracilis* (complexe), *Dasymys incomtus* et *Myomys daltoni.* Chez les eulipotyphles, les espèces *Crocidura olivieri* et *Atelerix albiventris* sont concernées [38,41,42,54,117-120]. Les populations de rongeurs sont étroitement liées au couvert végétal, où elles se déplacent et se nourrissent, ainsi qu’à la nature du sol dans lequel la plupart des espèces creusent des terriers pour s’abriter (Fig. 8) [59]. La petite souris domestique grise *M. musculus domesticus,* espèce envahissante majeure à l’échelle mondiale, trouve refuge dans les habitations, meubles et équipements domestiques [31,38,59]. Le rat indigène à mamelles multiples (10 paires) *M. erythroleucus,* présent dans tout le pays mais plus fréquent dans les zones sahéliennes, occupe à la fois les habitations péri-domestiques et les zones sauvages de savanes soudaniennes où il creuse des terriers plus ou moins profonds [43-45,59]. Le rat roussard *A. niloticus* habite principalement à proximité des habitations et dans les zones cultivées. Ne creusant pas de terriers, il utilise les clôtures de champs et les buissons touffus pour y installer son nid, et peut également occuper des terriers abandonnés [43-45,59]. Le rat noir commensal *R. rattus,* introduit au Sénégal par les marins portugais au 15^e^ siècle, a pénétré à l’intérieur du pays en utilisant les voies commerciales (fleuves et routes). Il fréquente les milieux sahéliens mais peut aussi être présent dans les zones soudaniennes. Le rat de Gambie ou rattoto *C. gambianus*, anthropophile est largement distribué dans les milieux sahéliens et guinéens.

Le mastomys du Natal ou rat à mamelles multiples du Natal *M. natalensis* est commun dans les zones sahéliennes et soudaniennes où il vit exclusivement à l’intérieur des habitations. Les musaraignes *C. olivieri,* au régime insectivore et carnivore, sont principalement trouvées dans les zones humides et à l’intérieur des habitations [43-45,59,115].

Chez les principaux réservoirs de *Borrelia* identifiés au Sénégal (Tableau I, Fig. 8), une prévalence de 9,2% de l’infection à *Borrelia* a été observée. Cette prévalence concerne à la fois des espèces saharo-sahéliennes, telles que *Gerbillus gerbillus*, *G. occiduus* et *G. tarabuli*, et des espèces soudano-sahéliennes, notamment *A. niloticus, M. erythroleucus* et *M. huberti* [42]. Des petits mammifères capturés dans la région de Fatick ont montré un taux de portage de 17,5% chez deux espèces de petits rongeurs, *A. niloticus* et *M. musculus domesticus* [88]. Un taux d’infection de 15,2% par *B. crocidurae a* été identifié chez cinq espèces de rongeurs: *M. musculus domesticus, G. nigeriae, Taterillus* sp., *A. niloticus* et *M. erythroleucus* [30]. Par ailleurs, une prévalence de 12% a été détectée spécifiquement chez les souris domestiques envahissantes, *M. musculus domesticus* dans la région de Saint-Louis (Fig. 8) [94]. Aucun cas d’infection à *B. crocidurae* n’a été rapporté chez le réservoir animal vertébré en dehors de l’aire de répartition géographique des tiques *O. sonrai* [38,117]. Les données recueillies au Sénégal, portant sur au moins 100 spécimens testés par espèce, révèle une prévalence globale d’infection à *Borrelia* variant selon les espèces. Une prévalence de 15,3% (139/906) a été observée chez *A. niloticus,* 14,7% (14/95) chez *Crocidura* sp., 11,5% (103/897) chez *Mastomys* sp., 5,7% (30/523) chez *M. musculus domesticus,* et 5% (5/100) chez *Gerbillus* sp. (Tableau I).

**Tableau I:** Distribution de la prévalence de l’infection à *Borrelia* chez des espèces de petits mammifères en fonction de la pluviométrie, du domaine climatique (DC) et de la localité, au Sénégal

Pluviométrie (mm)	DC	Région	Famille	Petits mammifères	I/T	%	Référence
50 – 250	Sahélien	Saint-Louis	Muridae	*Arvicanthis niloticus*	88/630	13,9%	38
				*Mastomys huberti*	37/235	15,7%	38
				*Mus musculus*	23/319	7,2%	38,94
				*Taterillus* sp.	1/10	10%	38
				*Gerbillus* sp.	1/20	5%	30
			Soricidae	*Crocidura* spp.	4/55	7,3%	38
250 – 500	Sahélo-soudanien	Dakar	Nesomyidae	*Cricetomys gambianus*	3/15	20%	38
			Muridae	*Arvicanthis niloticus*	4/18	22,2%	38
		Thiès	Muridae	*Mastomys erythroleucus*	24/136	17,6%	38
				*Arvicanthis niloticus*	13/94	13,8%	38
				*Gerbilliscus gambianus*	3/7	42%	38
		Louga	Muridae	*Mus musculus*	5/159	3,1%	38
				*Arvicanthis niloticus*	16/96	16,6%	30,38,72
				*Taterillus* sp.	10/53	18,8%	30,38,72
				*Gerbillus* sp.	4/80	5%	30
500 – 750	Soudanien	Fatick	Muridae	*Arvicanthis niloticus*	18/68	26,5%	38,88
				*Mastomys erythroleucus*	42/526	7,9%	38
				*Rattus* spp.	2/6	33%	38
				*Mus musculus*	2/45	4,7%	38,88
			Soricidae	*Crocidura* spp.	10/40	25%	38,88

Pluviométrie: Moyenne annuelle des précipitations de 1970 à 1999

I/T = Nombre de spécimens infectés (I)/Testés (T)

Precipitation: Average annual precipitation from 1970 to 1999

I/T = Number of specimens infected (I)/Tested (T)

## Climat et environnement

La distribution des tiques *O. sonrai* au Sénégal dépend des conditions climatiques liées à la fréquence de la pluviométrie, à la température, au type de sol ainsi qu’aux paramètres du sol favorables à leur implantation [37,40,117]. Dans la moitié ouest de l’Afrique de l’Ouest, la distribution des tiques *O. sonrai* apparaît indépendante des cours d’eau et d’autres facteurs environnementaux, tels que la végétation, la topographie, les zones montagneuses, l’activité agricole ou encore la densité de population. En revanche, dans la partie est de la région, notamment au Mali, la présence de *O. sonrai* est étroitement liée au réseau hydrographique, en particulier au fleuve Niger et à ses principaux affluents [117]. Les sols composés de sable fin associés à de l’argile à limon gros sont particulièrement plus adaptés à l’installation des tiques *Ornithodoros* [37,40,120]. Ce type de sol garde l’humidité et participe au maintien des foyers de la borréliose à tiques dans la nature. Les modifications des caractéristiques du sol peuvent avoir un impact négatif sur la survie des tiques dans leur environnement. Des études ont en effet montré l’absence du vecteur de la borréliose à tiques dans certaines zones localisées au sein de régions pourtant endémiques, suggérant que des conditions édaphiques défavorables peuvent limiter leur présence [37,42,85].

Les variations climatiques observées depuis 1970 au Sénégal liées au déficit pluviométrique, ajoutées à l’action anthropique ont entraîné des modifications remarquables sur les écosystèmes [120]. La persistance de la sécheresse en région subsaharienne a permis à la tique *O. sonrai* de coloniser de nouveaux territoires en zone soudanienne avec, en conséquence, l’extension vers le sud de l’aire d’endémie de la FRT [85,104,117,120]. L’étude de 66 stations météorologiques au Sénégal a révélé une extension de la répartition des tiques *O. sonrai,* corrélée à la pluviométrie et à la migration vers le sud de l’isohyète 750 mm en zone de savane soudanienne [37,117,120,121].

## Fièvre récurrente à tiques au Sénégal

C’est au Sénégal que la plupart des données relatives aux infections humaines à *B. crocidurae* ont été établies (Tableau II). La létalité attribuable à la FRT y est cependant mal connue [49,68,121], probablement d’environ 0,5%, mais elle reste inférieure à celle due à *B. duttonii* qui est comprise entre 2 et 5% en Afrique de l’Est, du Sud et Centre [55,56,78]. Un seul cas mortel a été rapporté dans la littérature [55] et un autre cas de FRT détecté chez une femme enceinte a été associé à une fausse couche en zone rurale [49]. Des études longitudinales sur la borréliose à tiques ont été menées dans la zone de Dielmo-Ndiop, située à 280 km au sud-est de Dakar, et à Niakhar dans la région de Fatick. La végétation et le climat de la zone correspondent à la savane soudanienne, et la population est composée d’un mélange d’ethnies wolofs et sérères, dont l’agriculture constitue l’activité principale. La surveillance médicale et épidémiologique de la population des villages de Dielmo-Ndiop a débuté en juin 1990 à Dielmo et en juillet 1993 à Ndiop. Les données épidémiologiques et cliniques sur la FRT ont été rapportées par plusieurs travaux de recherche [36,37,39,82,88,95,121]. Les patients atteints de FRT présentaient des symptômes non spécifiques dont une fièvre élevée (38 °C à 40 °C), des maux de tête (céphalées), des vomissements, de la diarrhée, des douleurs dorsales, des arthralgies, des courbatures, ou des douleurs abdominales [68,91,97]. Entre 2011 et 2016, les données recueillies au niveau du POC de Niakhar, dans la région de Fatick, ont montré que la FRT était la principale cause des motifs de consultation pour syndrome fébrile par structure sanitaire avec le paludisme et la grippe [1,111]. Une prévalence élevée de 11,7% y a été rapportée [88]. Les cas humains de FRT ont été enregistrés plus fréquemment entre juillet et septembre [36,88,121]. Dans la région de Thiès, une étude rétrospective d’une cohorte de patients fébriles, basée sur la détection des échantillons de plasma par la qPCR entre 2018 et 2019 a montré une prévalence de 15% [71].

**Tableau II:** Prévalence et/ou nombre de cas de fièvre récurrente à tiques enregistrés au Sénégal de 1928 à 2022

Année	Région	Zone	Méthodes de détection	Nombre de cas ou prévalence	Référence
1928	Dakar	Centre-ville	Goutte épaisse et frottis	1 cas d’infection spirochétienne	76
1932	Dakar	Centre-ville	Goutte épaisse et frottis	16 cas d’infection spirochétienne	77
1934	Dakar	Centre-ville	Goutte épaisse et frottis	46 cas de spirochétose	2
1942–1945	Dakar	Centre-ville	Goutte épaisse et frottis	198 cas de spirochétose	10
1946	Dakar	Centre-ville	Goutte épaisse et frottis	85 cas hospitalisés	9
1947	Dakar	Centre-ville et Medina	Goutte épaisse et frottis	92 cas hospitalisés	13
1983	Dakar	Centre-ville	Goutte épaisse et frottis	23 cas hospitalisés	6
1989–1990	Thiès	Keur Moussa	Goutte épaisse et frottis	4,2% des enfants de 10–14 ans	118
1991	Thiès	Diakhao Thialy	Goutte épaisse et frottis	10% de cas de borréliose	36
1990–1992	Fatick	Dielmo-Ndiop	Goutte épaisse et frottis	5,2% de cas de borréliose	119
1996	Fatick	Dielmo-Ndiop	Goutte épaisse et frottis	10% de cas de borréliose	119
1990–2003	Fatick	Dielmo-Ndiop	Goutte épaisse et frottis	11% de la population par an	121
2006	Fatick	Dielmo	Goutte épaisse et frottis	11% de cas de borréliose	121
2008–2009	Fatick	Dielmo-Ndiop	PCR sang capillaire du doigt	11% de cas de borréliose	34
2011	Fatick	Dielmo	PCR sang capillaire du doigt	13% de cas de borréliose	95
2010–2011	Fatick	Niakhar	PCR sang capillaire du doigt	19% de cas de borréliose	82
2010–2011	Louga	Keur Momar Sarr	PCR sang capillaire du doigt	6% de cas de borréliose	82
2010–2011	Fatick	Dielmo-Ndiop	PCR sang capillaire du doigt	9,7% de cas de borréliose/ 9. 7% of borreliosis cases	82
2011–2012	Fatick	Dielmo-Ndiop	PCR sang capillaire du doigt	10% de cas de borréliose	111
2013–2015	Fatick	Dielmo-Ndiop	CR sang capillaire du doigt	40 cas de borréliose	39
2016	Dakar	Pikine	Goutte épaisse et PCR	1 cas hospitalisé	35
2016	Fatick	Niakhar	PCR sang capillaire du doigt	11,7% de cas de borréliose/ 11. 7% of borreliosis cases	88
2011–2016	Fatick	Dielmo-Ndiop	PCR sang capillaire du doigt	4,8% de cas de borréliose	1
2019	Fatick	Niakhar & Touba-Couta	PCR sur *P. f* TDR	16% de cas de borréliose	87
2018–2019	Thiès	Thiès	PCR sang veineux	15,5% de cas de borréliose	71
2019	Thiès	Keur Moussa	PCR sur *P. f* TDR	6% de cas de borréliose	87
2019	Saint-Louis	Richard-Toll	PCR sur *P. f* TDR	4% de cas de borréliose/ 4% of borreliosis cases	87
2019	Kaffrine	Kaffrine	PCR sur *P. f* TDR	10% de cas de borréliose/ 10% of borreliosis cases	87
2018–2021	Diourbel	Touba	PCR sur sang	5,7% des pèlerins du Grand Magal de Touba/ 5. 7% of pilgrims at the Grand Magal of Touba	57
2020–2022	Sedhiou	Bounkiling	NGS sur plasma	2,4% de cas de borréliose	93
2020–2022	Thiès	Tivaouane	NGS sur plasma	6% de cas de borréliose	93
2022	8 régions testées au Sénégal	Régions endémiques	PCR sur *P. f* TDR	216 cas de borréliose 0,18% à 17,5% selon la localité	113

NGS: Next-generation sequencing

**Figure 9:**
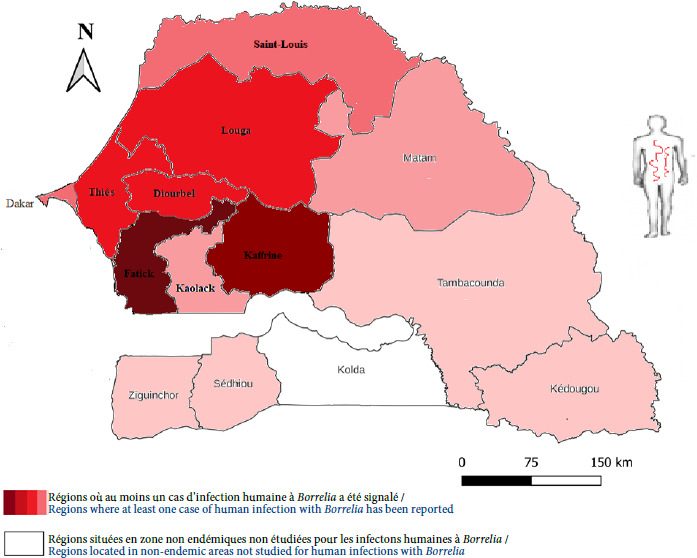
Distribution des cas humains autochtones de fièvre récurrente à tiques à *B. crocidurae* diagnostiqués au Sénégal, 1991-2023

Dans une étude innovante réalisée de janvier à décembre 2019, le dépistage par qPCR de *B. crocidurae* à partir de cassettes de test de diagnostic rapide (TDR) négatif pour *P. falciparum* dans quatre régions du Sénégal a indiqué que le taux d’infection chez les patients fébriles non palustres était de 7,22% [87]. La prévalence de l’infection à *Borrelia* la plus élevée était notée dans la zone soudano-sahélienne des régions de Fatick (16%) et de Kaffrine (10%), puis dans la zone sahélosoudanienne de la région de Thiès (6%). La plus faible prévalence était observée dans la zone sahélienne de la région de Saint-Louis (4%) [87], et les cas de FRT ont été observés en toute saison [1,36,87,88,111,121] (Fig. 9).

Des cas de FRT ont été détectés au Sénégal par des techniques de biologie moléculaire en dehors de l’aire de répartition connue de la tique vectrice *O. sonrai* et de celle des petits mammifères réservoirs trouvés naturellement infectés par *B. crocidurae* [26,38,80,93,117]. Il est probable que ces cas ne soient pas autochtones, mais plutôt importés, les patients fébriles ayant séjourné dans des zones endémiques du Sénégal ou d’Afrique de l’Ouest. Cela souligne l’importance de toujours investiguer la notion de voyage chez les patients fébriles présentant une fièvre récurrente d’origine inconnue.

**Figure 10:**
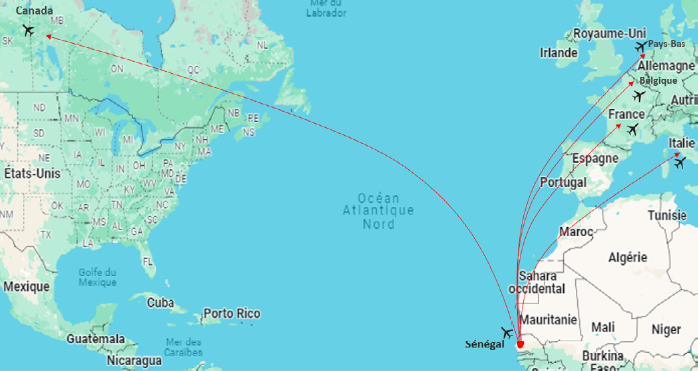
Distribution des cas de fièvre récurrente à tiques à *B. crocidurae* diagnostiqués chez des voyageurs revenant du Sénégal et des pays limitrophes, 1991-2021

Enfin, des cas de FRT ont été rapportés dans la littérature chez des voyageurs en provenance des zones endémiques africaines [65], et le plus grand nombre de cas identifiés provenait du Sénégal (Fig. 10). Entre 1990 et 2021,20 cas de FRT importés due à *B. crocidurae* ont été détectés, dont 14 en France, 2 en Italie, 2 en Belgique, 1 aux Pays-Bas et 1 au Canada (Fig. 10) [3,14,24-26,32,48,52,58,60,61,83,97,116].

### Diagnostic

Au Sénégal, comme dans les autres pays endémiques d’Afrique de l’Ouest, en l’absence de signes cliniques évocateurs d’une autre maladie, le paludisme a longtemps été considéré comme la première cause de fièvre [122]. Les symptômes de la FRT sont peu spécifiques, et longtemps confondus à ceux d’un paludisme parce que similaires [116]. Des cas de co-infections *Borrelia-Plasmodium falciparum* sont rarement diagnostiqués dans les structures sanitaires, du fait de l’absence de microscopistes bien formés pour identifier les deux pathogènes par la goutte épaisse (GE) de sang. Pourtant, au sein du laboratoire MINES (Maladies infectieuses, négligées et émergentes au Sud) de Dakar, des co-infections *Borrelia/Plasmodium* ont été régulièrement mises en évidence. Elles ont été détectées chez des patients fébriles de Dielmo, en utilisant la méthode classique de la goutte épaisse. Ces détections ont eu lieu avant l’avènement des techniques de biologie moléculaire plus sensibles, déployées dans les POC de Niakhar et Dielmo. Du fait de l’absence de signes cliniques spécifiques, il est indispensable d’établir un diagnostic différentiel de la borréliose à tiques [19,68,91,97].

La détection des *Borrelia* de la FRT est un réel défi en Afrique, à cause de l’absence de techniciens microscopistes bien formés, particulièrement depuis l’avènement des TDR. Le diagnostic de la FRT repose sur l’observation des spirochètes ou *Borrelia* lors de l’examen d’une goutte épaisse de sang ou d’un frottis sanguin coloré au Giemsa, une technique identique à celle utilisée pour détecter les hématozoaires du paludisme [41]. Cette technique présente une faible sensibilité et nécessite une expertise ainsi qu’un technicien averti, pour rechercher spécifiquement les *Borrelia* [41,95]. À titre d’exemple, sur 27 échantillons positifs à *Borrelia* détectés par qPCR, seulement 4 ont été identifiés comme positifs par la goutte épaisse réalisée en dispensaire. En revanche, 15 échantillons ont été reconnus positifs par des microscopistes bien qualifiés, également *via* la goutte épaisse. La technique d’inoculation intrapéritonéale de sang de patients fébriles ou d’organes suspects à la souris blanche demeure plus sensible que la goutte épaisse et le frottis sanguin [41]. Cependant, cette méthode n’est pas adaptée au diagnostic dans les établissements de santé.

Ces dernières années, l’apport des techniques de la biologie moléculaire pour la détection des spirochètes a été majeur. La PCR ou la qPCR sont les méthodes de diagnostic moderne les plus sensibles [95]. Ces techniques ont été mises en place par nos équipes, dans de petits laboratoires POC de proximité, installés en zone rurale au Sénégal, tout d’abord dans la station de recherche de Dielmo-Ndiop [111], puis à Niakhar. Ces POC ont permis de détecter des agents pathogènes responsables de fièvres d’origine inconnue et l’instauration de traitements thérapeutiques appropriés [111]. Le dépistage moléculaire de ces micro-organismes pathogènes dans les POC a contribué à mieux évaluer la prévalence et l’incidence de la FRT dans les zones endémiques du Sénégal [1,82,88,95,113]. Plus récemment, l’indentification des *Borrelia* par l’outil MALDI-TOF MS, a été appliquée sur des souches en culture ou pour différencier des tiques infectées ou non (Fig. 4) [20,50]. Enfin, une nouvelle technique de diagnostic spécifique pour la détection rapide des *Borrelia* de la FRT dans les régions endémiques à revenus limités a fait toutes ses preuves [64]. Il s’agit de tests d’amplification isothermique en boucle (LAMP) que l’on peut utiliser dans les POC installés en zones rurales [64,92]. La technique LAMP repose sur l’utilisation de six amorces spécifiques et des réactifs nécessaires à leur amplification, pendant 60 minutes. La grande sensibilité et la spécificité de ce test, ainsi que la simplicité de la procédure d’extraction de l’ADN font de la technique LAMP un outil fiable et efficace pour diagnostiquer la FRT [64].

### Traitement

Comme les autres agents responsables de FRT, *B. crocidurae* est sensible à de nombreux antibiotiques facilement disponibles en pharmacie. Le traitement antibiotique de choix pour la FRT est la doxycycline. Chez l’adulte, la posologie recommandée est de 100 mg deux fois par jour. Pour l’enfant, la dose est de 4 mg/kg en une seule prise [68]. L’érythromycine a été utilisée chez les femmes enceintes et les enfants de moins de huit ans [16,68]. Concernant l’azithromycine ou l’amoxicilline, les preuves concernant leur efficacité sont limitées. Dans les formes sévères ou neurologiques de FRT ou en cas de vomissements, la ceftriaxone en intraveineuse et/ou intramusculaire en fonction du stade de la maladie est une option thérapeutique de choix [12,67]. Les *Borrelia* sont résistantes à la rifampicine, au métronidazole et aux sulfamides [68].

## Lutte contre la fièvre récurrente à tiques au Sénégal

Les méthodes classiques de lutte contre les maladies à transmission vectorielle reposent souvent sur l’utilisation d’insecticides et/ou d’acaricides chimiques par la pulvérisation ou le saupoudrage afin de réduire ou combattre le vecteur. Ces méthodes classiques présentent des risques de contamination pour les populations rurales exposées, souvent mal informées des risques liés à leur utilisation. À cela s’ajoutent les contraintes écologiques spécifiques aux tiques ornithodores qui vivent à l’intérieur des terriers (endophiles), et les problématiques de résistance. C’est la raison pour laquelle une stratégie alternative non chimique a été développée pour lutter contre la borréliose à tiques en zones endémiques au Sénégal.

De 2013 à 2015, elle a été mise en place à Dielmo et Ndiop [39]. Dans les zones rurales africaines, l’habitat traditionnel des maisons est en terre battue avec un sol non cimenté, parfois cimenté mais souvent dégradé [39]. Cette stratégie visait à cimenter le sol des chambres de couchage, des greniers et de la cuisine. L’objectif principal était d’éliminer les terriers de petits rongeurs, afin de réduire les risques de contact avec la tique vectrice [39]. La stratégie ciblait trois actions principales: premièrement, éliminer le contact humain-tiques en remblayant tous les terriers et anfractuosités au sol avec un mélange de ciment et d’argile, sur une épaisseur d’environ 5 cm. Deuxièmement, surveiller de façon régulière et systématique l’apparition de nouveaux terriers et crevasses, pour les refermer rapidement [39]. Troisièmement, capturer les petits mammifères à l’aide d’une colle liquide non toxique, afin de réduire leur présence dans les habitations et ainsi limiter la propagation des tiques. Parallèlement, une sensibilisation à l’hygiène sanitaire a été menée, notamment par le balayage régulier de l’intérieur des habitations, pour éliminer les tiques errantes à l’extérieur des terriers et réduire le risque de contact humaintique [39]. Ces mesures, mises en œuvre avec l’implication collective des populations, ont permis une réduction significative de l’incidence de la fièvre récurrente à tiques. Ainsi, à Dielmo, l’incidence a diminué de 89,8%, passant de 10,55 à 2,63 cas pour 100 personnes-année. À Ndiop, la baisse a été de 81,5%, de 3,79 à 1,39 cas pour 100 personnes-année [39]. Au total, 36 cas d’infection de borréliose ont été évités, pour un coût économique de 526 €, soit 345 033 FCFA par infection évitée. Depuis janvier 2016, cette stratégie et ses mesures d’accompagnement ont été transférées au niveau communautaire [39]. La dotation en matériel (colle liquide et ciment) a continué d’être fournie pour capturer les petits mammifères et refermer les terriers dans les habitations. En 2017, cette dotation a été interrompue, mais les populations se sont définitivement approprié la stratégie de lutte préventive [39]. Aujourd’hui, dans ces deux villages, les cas humains de borréliose sont devenus rares, voire absents.

## Conclusion

Dans un contexte de pré-élimination du paludisme, la FRT représente le premier motif de consultation pour syndrome fébrile dans de nombreuses structures sanitaires en zones rurales endémiques du Sénégal. Les études menées dans ce pays sur la borréliose à tiques au cours des 30 dernières années montrent que cette maladie doit être une préoccupation sanitaire en milieu rural. Cependant, la FRT reste méconnue des cliniciens et des autorités. Elle reste peu suspectée et rarement diagnostiquée dans les causes de fièvres, en dehors des centres pilotes où des laboratoires POC sont installés. Cette revue s’adresse aux décideurs et autorités sanitaires, tant nationaux qu’internationaux. Elle appelle à un plaidoyer fort pour une meilleure prise en compte de cette maladie, qui reste un problème de santé publique négligé. Les deux tiers nord du territoire national, soit dix régions (Dakar, Thiès, Diourbel, Louga, Saint-Louis, Matam, Fatick, Kaolack, Kaffrine et Tambacounda), sont endémiques, caractérisées par une présence massive des tiques vectrices *O. sonrai,* infectées par *B. crocidurae,* dans la nature et les habitations humaines. De plus, on y observe une fréquentation constante des petits mammifères réservoirs de l’infection à l’intérieur des maisons, ce qui favorise la persistance de la maladie. Cette situation souligne l’importance d’intégrer la fièvre récurrente à tiques (FRT) due à *B. crocidurae* dans la liste des maladies tropicales négligées (MTN). Il est également crucial de mettre en place un programme de surveillance épidémiologique de cette maladie. Enfin, une sensibilisation de masse des populations vivant dans les régions endémiques doit être organisée pour mieux prévenir et contrôler la borréliose. (Fig. 9). La stratégie de lutte préventive mise en place à Dielmo et Ndiop, accompagnée de mesures d’appui, a permis une réduction significative de l’incidence de la maladie. Cette réussite est due à l’implication et à l’adhésion des populations locales. Cette approche mérite d’être étendue à grande échelle dans toutes les régions rurales endémiques du Sénégal et de l’Afrique de l’Ouest. Elle pourrait s’inscrire dans un programme national, voire sous-régional, visant à limiter les pertes économiques liées à l’absentéisme des malades et aux prises en charge inadaptées des fièvres non palustres.

Enfin, pour une meilleure prise en charge rapide et efficace des cas de FRT au Sénégal, il serait idéal d’implanter des POC dans les régions endémiques en priorité, puis dans les autres régions. Cela permettrait un suivi plus précis et un contrôle de la survenue des cas de FRT, notamment chez les patients consultant pour fièvre non palustre. Enfin, une réflexion avancée porte actuellement sur la mise au point d’une cassette de test rapide (TDR) spécifique à *B. crocidurae.* Ce dispositif, simple d’utilisation et accessible partout, faciliterait le diagnostic rapide et améliorerait la prise en charge des cas de FRT dans les principaux foyers endémiques du Sénégal, ainsi que dans d’autres pays d’Afrique de l’Ouest et du Nord.

## Remerciements

Les auteurs remercient Jean-François Trape et Didier Raoult pour leur aide précieuse dans la recherche d’informations, de publications et d’articles scientifiques anciens non disponibles en ligne, ainsi que pour leur contribution à la connaissance de l’épidémiologie de la FRT au Sénégal et, plus largement, pour l’ensemble de leurs travaux sur cette maladie.

## Financement

Cette étude a été soutenue par l’Institut hospitalo universitaire (IHU) Méditerranée Infection, l’Agence nationale de la recherche (ANR) dans le cadre du programme « Investissements d’avenir », référence ANR-10-IAHU-03, et la région Provence-Alpes-Côte d’Azur.

## Déclaration du comité d’examen institutionnel

Le Comité national d’éthique pour la recherche en santé du Sénégal (CNERS) a approuvé cette étude dans le cadre du protocole « Agents pathogènes responsables des fièvres (IDEPATH) » SN21/09 SN37/09 en 2012, sous le numéro 00081MSAS/DGS/DS/CNERS.

## Déclaration éthique

Les auteurs déclarent que toutes les données ont été collectées, traitées et analysées conformément aux réglementations éthiques et aux lois en vigueur en matière de protection des données personnelles. Toutes les informations permettant d’identifier les participants à l’étude ont été anonymes afin de garantir leur confidentialité et leur vie privée, conformément aux directives institutionnelles et aux protocoles approuvés pour le traitement des données sensibles. Les sources des données utilisées ont été correctement citées dans le manuscrit.

## Contribution des auteurs

NDIAYE El Hadji Ibrahima: Collecte des données, méthodologie et rédaction.

DIATTA Georges: Méthodologie, validation des données.

SOKHNA Cheikh: Conceptualisation, méthodologie, validation des données PAROLA Philippe: Conceptualisation, méthodologie, validation des données et supervision

## Conflits d’intérêts

Les auteurs déclarent n’avoir aucun conflit d’intérêts.
