# Supplementary material for: West African tick-borne relapsing fever, a neglected disease of major public health importance in Senegal
Source: Med Trop Sante Int. 2025 Sep 24;5(4):mtsi.v5i4.2025.573. [Article in French] doi: 10.48327/mtsi.v5i4.2025.573 (PMC12957993; doi:10.48327/mtsi.v5i4.2025.573)
Supplement: Related texts

# Research article: West African tick-borne relapsing fever, a neglected disease of major public health importance in Senegal

## Introduction

*Borrelia* are spirochete bacteria that cause borreliosis, a widespread zoonotic disease [27,29,118,119]. There are three main groups of diseases caused by *Borrelia* bacteria [11,96]. The first group corresponds to the bacteria responsible for Lyme disease, also known as Lyme borreliosis. This group includes approximately 20 described species worldwide. The most frequently implicated in human disease are *B. burgdorferi sensu stricto, B. garinii,* and *B. afzelii.* These species are transmitted by hard ticks belonging to the genus *Ixodes* [11,96]. The second group includes *Borrelia* species that cause relapsing fevers, which are mainly transmitted by soft ticks [68,119]. The third group corresponds to relapsing lice fever, which is caused by *Borrelia recurrentis* and transmitted by body lice *(Pediculus humanus humanus)* and possibly head lice *(P. h. capitis)* [15,17,66,100].

The clinical forms of TBRF can be mild or severe [29,68]. More than 14 species of *Borrelia* that cause tick-borne relapsing fever are known worldwide. In eastern and southern Africa, TBRF caused by *Borrelia duttonii* is well known and associated with significant case fatality rate (CFR). In Tanzania, it is one of the top 10 causes of death in children under five years of age, with a perinatal CFR of 436 per 1,000 in endemic regions [28,73,79].

It is transmitted by soft ticks belonging to the *Ornithodoros moubata* group, specifically *O. moubata* and *O. porcinus* [29,84]. In West and North Africa, the agents of TBRF are *Borrelia crocidurae, Borrelia hispanica,* and *Borrelia merionesi.* These pathogens are mainly transmitted by several species of soft ticks: *Ornithodoros sonrai, O. marocanus, O. merionesi,* and *O. costalis* [117]. *Borrelia hispanica* is found in coastal areas of North Africa [29,117]. Finally, *B. crocidurae* is more widespread in West Africa and is also found in North Africa [29,117].

Small wild mammals are the main reservoirs of the *Borrelia* species that cause TBRF [38,117,118], with the exception of *B. duttonii*, for which humans are the main reservoir [28].

In Senegal, *B. crocidurae* is the only known agent of TBRF. It is transmitted by *O. (Alectorobius) sonrai* ticks that live in the burrows of small wild and semi-domestic mammals [87,117,118]. In rural, endemic areas of Senegal, TBRF accounted for 11% of consultations for febrile syndrome from 1990 to 2003, with a prevalence ranging from 13% in 2009 to 12% in 2016 [1,88,95,113,121].

*Borrelia crocidurae* was first detected in Senegal in 1917 in the blood of a shrew [69].

The role of the tick *O. sonrai* was suspected in the 1930s as a vector of TBRF (formerly known as Dakar relapsing fever or Dakar spirochetosis) [77]. Interest in the disease and its epidemiology renewed in the 1990s [36,54,118,121], when TBRF cases were often clinically confused with malaria cases [4,36,118]. The establishment of small point-of-care (POC) molecular diagnostic laboratories in rural Senegal has allowed for the early diagnosis and treatment of fevers of unknown origin [111]. However, more than 100 years after its discovery, TBRF remains poorly understood by health workers and populations in Senegal [39,88]. Human cases are still rarely diagnosed due to a lack of expertise and/or accessible, simple diagnostic tools [111]. Therefore, confirmed human cases are most often reported in studies identifying causes of fever of unknown origin, and the disease remains neglected in Senegal [1,71,87,88,93,111]. The aim of this narrative and systematic review is to summarize the current knowledge of the clinical, entomological, and mammalogical data on TBRF in Senegal. The goal is to propose modern, simple, and appropriate diagnostic methods and programs for the adoption of control strategies in endemic regions.

## Data collection methodology

Starting on October 16, 2023, we conducted a systematic literature search in several databases, including Medline, PMC, PubMed, Google, and Google Scholar, as well as relational resources and university libraries to access older publications not available online. The search was conducted using the following terms: “ticks” OR *“Ornithodoros* ticks” OR *“Ornithodoros senegalensis”* OR *“Ornithodoros sonrai”* OR *“Borrelia”* OR *“Borrelia senegalensis”* OR *“Borrelia recurrentis”* OR specific species names of *Borrelia,* such as *“Borrelia crocidurae”* etc.; and “relapsing fever” OR “tick-borne relapsing fever” OR “West African tick-borne relapsing fever” OR “tick-borne relapsing fever in Senegal.” These terms were adapted to the search format of each database.

After removing duplicates using Zotero (version 6.0.28) and manually sorting the results, we preselected publications based on their titles and abstracts. Articles that did not address TBRF or were unrelated to the objectives of our study (epidemiology, mammalogy, entomology, transmission, vectors, reservoirs, carriage of infectious agents, clinical features, diagnosis, and treatment of TBRF in West Africa) were excluded. We then conducted a comprehensive review of the remaining 375 publications. Articles that did not meet the inclusion criteria (focusing on TBRF in West Africa, particularly Senegal) were eliminated from the analysis. We also excluded publications that we were unable to obtain (Fig. 1).

During our review of the texts, we examined the reference lists of the articles to identify other relevant publications that were not included in the initial search. Of the 123 eligible articles, we extracted the following information: first author; title; year of publication; type and location of study; study period; location of acquisition/infection with *Borrelia; Borrelia* species; tick vector species; percentage of ticks or vectors infected with *Borrelia;* small mammal reservoirs infected or not infected with *Borrelia;* diagnostic method (microscopy, serology, or molecular diagnosis); intraperitoneal inoculation in adult albino white mice *(Mus musculus,* SWISS strain), degree of diagnostic certainty, number of patients, median and range of age, sex, symptoms, number of fever relapses, pregnancies, complications, treatments used and treatment regimens, number of patients treated or not treated, and means/methods of TBRF control.

**Figure 1:** Flow chart of the process of selecting relevant literature for systematic review

## History of tick-borne relapsing fever in Senegal

The TBRF agent was first visualized and described in 1917 in the blood of a small mammal of the order Eulipotyphla: a shrew initially identified as *Crocidura stampflii.* This shrew was trapped in the sewers of Dakar [69]. As part of his research into the role of animals as reservoirs of parasitic diseases, André Léger observed a spirochete whose description differed from that of other known bacteria. Its distinctive feature was the formation of chains composed of three to six spirochetes arranged end to end or in tangled clusters. Each organism viewed under a microscope appeared as a filament coiled in spirals and ending in tapered ends without visible cilia or flagella. The spirochete had four or five undulations on the same plane and measured approximately 14 to 16 μm in length and 2 to 5 pm in width [69]. Therefore, he proposed naming this spirochete *“Spirochaeta crocidurae*’, which was later renamed *“Borrelia crocidurae”.*

In 1920, human blood spirochetosis was reported in Dakar [90], and in 1923, it was found that rodents were infected with blood-borne spirochetes [70]. Through experimental infections in laboratory animals, Mathis (1926) demonstrated that two human subjects had been infected with spirochetes from rats captured in Dakar. Thus, he established that the blood-borne spirochetes identified in shrews could be pathogenic to humans. The symptoms observed in infected subjects were identical to those of “human relapsing typhus” [74]. At that time, there was an endemic outbreak of this disease in Dakar, the cause of which was unknown [75]. Mathis then demonstrated the similarity between human blood-borne spiro chetosis and those observed in small mammals. Mathis also highlighted the role of shrews and various small murid rodents as reservoirs of the pathogen responsible for human relapsing typhus. He proposed designating this endemic form observed in Dakar as Dakar tick-borne relapsing fever (TBRF) [76,77]. In the 1930s, a soft tick of the *Ornithodoros erraticus* complex was identified as the agent responsible for transmitting TBRF to humans [46,77]. These ticks were infected with a spirochete identical to those found in subjects with Dakar spirochetosis and in the blood of shrews and small murid rodents [76].

In 1949, Boiron stated that the rate of Dakar TBRF contamination by ticks of the *O. erraticus* complex depended on the type of housing and soil [13].

In the late 1940s, colonial doctors in West Africa began to pay attention to human infections caused by *Spirochaeta crocidurae,* the agent of Dakar TBRF. Clinical observations increased, describing the various forms and complications of the disease [10]. The most notable feature was the appearance of the temperature curve. This anomaly was accompanied by hepatic-vesicular, icteric, renal, and neurological manifestations, including pure meningeal forms, ocular disorders, and peripheral and medullary paralysis [10]. The disease was characterized by Larrey’s relapsing fever syndrome, which is defined by periodic episodes of fever separated by periods without fever, hence the future name TBRF given to the disease [101]. Based solely on the recurrence of fever in a patient in an endemic area, Bergeret and Raoult recommended seeking the spirochete responsible for Dakar TBRF [9,10]. At the same time, they established the therapeutic basis for treating patients with molecules such as novarsenobenzole, sulfarsenol, orsanine, tryparsamide, acetylsalicylic acid, diamidine, and penicillin, which produced satisfactory results [10]. In Dakar, the number of annual hospitalizations increased from 26 in 1942 to 86 in 1946 [9,76,77]. Thus, clinical and epidemiological knowledge about TBRF in West and North Africa largely came from doctors and hospitals in Dakar [10].

After a long period of declining research activity on TBRF, and even oblivion, renewed interest in this disease emerged in the 1990s. New studies began with population surveillance using thick blood smears. Between 1989 and 1990, *B. crocidurae* infections were detected in 0.9% of children under 15 years of age attending the Keur Moussa health center in the Thies region [118]. In this region, a prevalence of *B. crocidurae* of 4.2% was detected by thick smears in febrile patients examined there [118]. From June 1990 to May 1992 in Dielmo, a village in the Fatick region, the average annual incidence rate of TBRF was 5.2% among patients presenting with fever [36,120]. Depending on the year, clinical and parasitological follow-up of the village population of Dielmo, carried out over 14 years, showed that on average 11% of the population developed the disease, with the annual incidence rate of TBRF fluctuating between 4% and 25% [121]. The identification of *Borrelia* infections has been made easier with the introduction of POC molecular tools, a small diagnostic laboratory based on PCR or qPCR molecular biology analyses [111]. Thus, in 2010, the prevalence of TBRF was estimated at 13% by molecular biology analyses, whereas thick blood smear tests had only detected *Borrelia* in 2% of febrile patients [95].

Over the past 30 years, our knowledge of TBRF in Senegal has become more precise. This applies to several areas, including bacteriology, epidemiology, clinical aspects, entomology, and mammalogy [4,68,83,87,88,117]. TBRF was likely the second most common reason to seek medical care for vector-borne diseases after malaria. Currently, in the context of increased malaria control efforts, TFT is the primary vector-borne disease in certain rural, endemic areas of Senegal [27,29,118,120]. In September 2022, the monthly incidence of tick-borne borreliosis cases in the Niakhar area of Fatick ranged from 11 to 23, with a prevalence of 12%. A mass awareness campaign was conducted among the population, providing information on ways to combat the disease. Thanks to this intervention, the monthly incidence of human cases decreased in 2023 to only one to five cases per month out of an estimated population of more than 45,000 people (Diatta G, unpublished data) [33]. It was during these information and awareness-raising meetings, which brought together all the village chiefs as well as administrative and health authorities, that the constituent assembly took an important decision. The assembly assigned the name “Sibiru Magare Ndiock” to the TBRF for infection with *Borrelia* in mice, or small rodents, in the Serer language.

### Borrelia crocidurae

This bacterium was formerly known as *“Spirocheata crocidurae”* [69,110], and was officially renamed *Borrelia crocidurae* by Davis [110]. *Borrelia* are spiral-shaped bacteria (spirochetes) that are wavier than spiral. They measure 3 to 25 μm in length and 0.2 to 0.5 pm in width [47]. They are morphologically identical, with slender bodies and tapered ends (Fig. 2A and 2B). *Borrelia* lack true flagella but have 7 to 30 endoflagella in the periplasmic space. These endoflagella form a flexible helix that ensures their characteristic mobility [107]. These organisms have a genome consisting of a linear chromosome ranging in size from 900,000 to 920,000 base pairs (bp), as well as several circular and linear plasmids. Some species have up to 20 different plasmids [107]. All *Borrelia* have the same life cycle. They are extracellular and live in liquid environments, such as plasma or the hemolymph of arthropod vectors, or in the intercellular spaces of tissues [7,53]. These spirochetes can be detected in tissues after silver impregnation. They react to Giemsa staining, which gives them a purple or blue-violet color [37]. *B. crocidurae* can be cultured *in vitro* on modified Kelly medium [32,95,117].

The genome of *B. crocidurae* (1,557,560 bp, 27% GC) has been published [62]. Analysis of concatenated partial sequences of *Borrelia* in *Ornithodoros* ticks has contributed to the study of the phylogenetic relationships and geographical distribution of *B. crocidurae* in West Africa (Fig. 3A and 3B) [117]. Matrix-assisted laser de-sorption/ionization time-of-flight mass spectrometry (MALDI-TOF MS), a technology that has become a benchmark for the rapid, reliable, and low-cost identification of bacteria, was used to identify *Borrelia.* This method is based on gently ionizing proteins with a matrix and then separating them according to their mass-to-charge ratio in a time-of-flight analyzer. This generates specific protein profiles for each species, facilitating discrimination (Fig. 4A) [16,20]. This tool has also made it possible to distinguish between *O. sonrai* ticks that are infected and uninfected with *B. crocidurae* (Fig. 4B) [50].

**Figure 2A:** Visualization of *Borrelia crocidurae* grown at the IHU Méditerranée Infection laboratory in Marseille: indirect immunofluorescence (A): mouse serum, (x40 UV filter objective) and Gimenez staining (B): (x1000 immersion objective)

**Figure 2B:** Visualization of *B. crocidurae* under the microscope by Giemsa staining (C): amplified in the blood of white mice, albino variety, SWISS strain, (x1000 immersion objective); glutaraldehyde fixation (D&E): strains from the IHU Méditerranée Infection in Marseille, SU5000 BSE-ALL scanning electron microscope

**Figure 3A:** Phylogenetic relationships among *Borrelia* species using partial Intergenic spacer (IGS, 510 nucleotides) and partial FlaB gene (FLA, 269 nucleotides) concatenated sequences (PhyML 100 bootstraps, available at http://mobyle. pasteur.fr/cgi-bin/portal.py). The colored triangle estimated the *Borrelia* species diversity. Trape *et al.,* 2014, included in the *B. merionesi* clade (9 seq.) a *Borrelia* detected in a rodent captured in El Argoub (Morocco). The colored full circle correspond to the tick species determined by the 16S phylogenetic analysis of this study. The phylogram was constructed using a maximum-likelihood method from concatenated sequence data (220 sequences including GenBank reference sequences, 779 nucleotides). Bootstrap values >70 are shown (Scale bar, 0.01 substitutions per site). Seq. A.: *B. crocidurae* (GU350723 and NC017808), seq. B.: *B. recurrentis* (DQ000277 and DQ346814), seq. C.: *B. duttonii* (DQ000279 and DQ346833), seq. D.: *B. merionesi* (JX257047 and JX257050), seq. E.: *B. hispanica* (GU350718 and GU357614) and concatened sequences were used as references. doi: 10.1371/journal.pone.0078473.g004 from Trape *et al.,* 2013

**Figure 3B:** Phylogenetic relationships among Ornithodoros species using 16S rRNA sequences (820 seq.). The colored triangles indicate the genetic diversity of the sequences detected for each *Ornithodoros* species. The colored-spots correspond to the *Borrelia* species detected in each tick species. The phylogram was constructed using a maximum-likelihood method from partial 16S sequence data (457 nucleotides). Bootstrap values >90 are shown (Scale bar, 0.05 substitutions per site). *Ornithodoros moubata* (GenBank accession number AB073679), *O. porcinus* (GenBank acc. no. AB105451), *O. turicata* (GenBank acc. no. L34327), and *O. parkeri* (GenBank acc. no. EU009925) were treated as outgroups. Five of these species are newly described: *O. occidentalis, O. costalis, O. rupestris, O. kairouanensis,* and *O. merionesi.* doi: 10.1371/journal.pone.0078473.g002 from Trape *et al.,* 2013

**Figure 4A:** MALDI-TOF MS analysis of the reference strains of four *Borrelia* spp. and field isolates of both human and tick origin. spectra obtained by MALDI-TOF MS analysis of the reference strains of four *Borrelia* spp. (B. *hermsii* HS1 in green, *B. japonica* F63B in yellow, *B. afzelii* 103469 in red, *B. burgdorferi* ss B31 in blue) and field isolates of both human (B. *burgdorferi* ss UCSC and *B. garinii* G1) and tick origin (B. *burgdorferi* ZS7 and *B. lusitaniae* MT 0407-M8), all in black. doi: 10.1371/journal.pone.0088895.g001 from Calderaro *et al.,* 2014

**Figure 4B:** A 6-peak pattern for the specific detection of *Borrelia crocidurae* in *O. sonrai* ticks. doi: 10.1371/journal. pntd.0002984.g002 from Fotso Fotso *et al.,* 2014

### *Ornithodoros sonrai* and transmission

Ticks of the genus *Ornithodoros* belong to the Argasidae family, also known as soft ticks. Their thick, unsegmented, oval-shaped bodies consist of two regions: the idiosoma and the capitulum, which is located in the ventral position [36]. They have no eyes. Adults differ from nymphs mainly by the ventral position of the capitulum. In the larval stage, the capitulum is separated from the idiosoma and located at the terminal end [103]. The capitulum is located in a cavity of the idiosoma called the camerostome. The capitulum bears sensory organs (palps) and mouthparts (hypostome and chelicerae). The camerostome has lateral expansions called cheeks [36]. The idiosome’s integument is covered with small tubercles called mammillae [36]. On the dorsal side, the cuticle has flat areas where the mammillae are absent, corresponding to the insertions of the dorsoventral muscles [36]. The idiosoma bears the ventral insertion of the legs, as well as the genital and anal orifices. The legs (four pairs in adults and nymphs, and three pairs in larvae) are inserted directly into the tegument by a fixed part called the coxa [63,103] (Fig. 5).

The small tick *O. sonrai* (3 to 5 mm in adults) is classified within the *O. erraticus* complex, which currently comprises nine genetically distinct and morphologically similar species [106,117,121]. *Ornithodoros sonrai* ticks, like other ornithodores, are obligate ectoparasites. They are endophilic living in the wild by colonizing the burrows of small mammals, which provide them with microclimate conditions favorable to their development [103,117]. There are three stages—larval, nymphal, and adult—that undergo several stages of metamorphosis, sometimes called “moults,” during their development cycle [36,103,119].

The development cycle of *O. sonrai* has two phases: a parasitic phase on the rodent host and a free phase on the ground (Fig. 6). The parasitic phase is brief and involves a large number of blood meals. Adults feed on blood before each mating [102]. *Ornithodoros* ticks bite painlessly, usually at night while the host is sleeping in traditional dwellings with earthen floors frequented by rodents. The coxal fluid released during the meal is the source of *Borrelia* transmission [101]. After their meal, which lasts about 10 to 20 minutes, the ticks abandon the host to continue their life cycle on the ground [103]. Female *O. sonrai* lay eggs on the ground in burrows after a maximum of 13 days. In tropical areas, adult *O. sonrai* ticks reproduce throughout the year. *Ornithodoros* ticks have a long lifespan (between 10 and 20 years, depending on the species, temperature, and relative humidity), which explains their presence in abandoned rodent burrows [38,103,119]. *Ornithodoros sonrai* can ingest *Borrelia* (Fig. 6) during their blood meal from an infected vertebrate, most often small mammals, which are the main reservoirs of bacteria [68]. The ingested *Borrelia* develop in contact with the intestinal mucosa and then actively migrate to the salivary and coxal glands [51,107]. The infection is transmitted to vertebrates during a blood meal via secretions from the salivary and coxal glands [21,107]. The passage of *B. crocidurae* from the tick *O. sonrai* to the main vertebrate reservoir hosts, and vice versa, is necessary for the spread of the pathogen *B. crocidurae* and the establishment of endemicity [38,88,117]. There is also transovarian and transstadial transmission of *Borrelia* in ticks. During this process, the offspring of the infected female *O. sonrai* become infectious themselves. The infection is also transmitted from one stage of development to another, ensuring the maintenance of the *B. crocidurae* pathogen in *O. sonrai* ticks (Fig. 6). Therefore, ticks can also act as reservoirs for bacteria. *Borrelia* can remain inside ticks for several years without losing their pathogenicity [68,112]. The longevity of *Ornithodoros* ticks and maintenance of vector potential has been demonstrated in *Ornithodoros tholozani* ticks, which can transmit *Borrelia* after 11 years [98]. However, transmission in ticks may be accompanied by a gradual decrease in bacterial load over time [51,107].

**Figure 5:** Details of the dorso-ventral morphological features of an *O. sonrai* tick from Senegal under the scanning electron microscope

**Figure 6:** Circulation of *B. crocidurae* in the parasite cycle of the *O. sonrai* tick and accidental transmission of tick-borne relapsing fever to humans

### *Ornithodoros sonrai* and associated microorganisms in Senegal

In Senegal, *O. sonrai* colonizes the burrows of small rodents in the Saharan and Sahelian regions, as well as some located north of the 750 mm isohyet in the Sudano-Sahelian region [117]. Its southern distribution limit is at the SenegalGambia border, corresponding to the 750 mm isohyet (Fig. 7) [117]. The tick is absent in regions where rainfall exceeds the 750 mm isohyet, particularly in Ziguinchor, Kolda, Sédhiou, Kédougou, and the southern part of the Tambacounda region. This area corresponds to approximately latitude 13°15’N [117]. *Borrelia crocidurae* has been found in *O. sonrai* ticks collected in the northern two-thirds of Senegal [10,37,77,117,120]. Molecular biology tools have made it possible to refine the geographical distribution of *B. crocidurae* in *O. sonrai* ticks collected in endemic areas of Senegal (Fig. 7) [42,88,117]. In addition to its role as a vector for *B. crocidurae* infection, *O. sonrai* can harbor other microorganisms, such as *Coxiella burnetii,* the agent of Q fever, as well as bacteria of the genera *Bartonella, Wolbachia, Ehrlichia,* and *Anaplasma.* It also harbors *Occidentia massiliensis,* a member of the Rickettsiaceae family whose pathogenicity remains unknown [80-82,86].

### *Borrelia crocidurae* in humans

The development cycle of *Borrelia* begins with the active multiplication of bacteria in the vascular compartment. *Borrelia* have a preferential tropism for the vascular endothelium, central nervous system, liver, spleen, and bone marrow; however, the mechanism has not yet been elucidated [19,99,105]. *Borrelia crocidurae* can aggregate with erythrocytes, forming aggregates called erythrocyte rosettes. These structures disrupt the interaction between immune cells and bacteria, delaying the immune response. Additionally, the presence of these aggregates can cause microembolisms, resulting in tissue damage [18,109]. Furthermore, *B. crocidurae* shares with *Borrelia* of the *B. burgdorferi* complex the ability to activate vascular endothelial cells and promote transendothelial migration of neutrophils [109]. Together, these events lead to vascular damage and inflammatory reactions, forming the basis of the pathophysiology of TBRF caused by *B. crocidurae. Borrelia* are most often visible in peripheral blood during periods of fever. They disappear or become rare during periods of normothermia, and fever recurrence corresponds to a recurrence of bacteremia [5]. These *Borrelia* can modify their surface antigenic structure to temporarily evade the immune system [5]. The specific antibodies that appear during the first bacteremia episode destroy almost all of the *Borrelia* present in the blood [89]. However, these antibodies are only active for one recurrence and cannot target antigens of new surface proteins [8]. This phenomenon of immune escape is responsible for bacterial recurrences. The average incubation period is seven days, and the disease begins with chills followed by a 40°C fever [47,123].

**Figure 7:** Distribution of *B. crocidurae* detected in *O. sonrai* ticks from 1991-2022 in Senegal

Fever episodes may be accompanied by headaches, joint pain, muscle pain, bone pain, and digestive disorders such as abdominal pain, vomiting, and diarrhea [68,91,97]. Hepatosplenomegaly and anemia may also be observed [68,91,97]. After a feverish period of 3 to 4 days, the episode ends with a rapid drop in temperature, sweating, and a drop in blood pressure [47,68,97]. This is followed by a fever-free period of 2 to 10 days during which patients continue to experience muscle and bone pain [47,68]. Untreated patients may experience up to nine recurrences over several months, with fever episodes occurring every few days [9,47]. Neurological signs generally appear during the second fever episode [19]. Complications such as meningoencephalitis, hepatorenal syndrome, eye damage, and spontaneous abortions in pregnant women can occur at any time during the course of the disease [10,23,49,55,56,68,102]. However, facial paralysis, which is often observed as a main sign of neuroborreliosis caused by species of the *B. burgdorferi sensu lato* (sl.) complex, has not been observed with *B. crocidurae* [19]. In practice, the clinical picture is often that of “fever of unknown origin,” which is well known in tropical areas.

### The natural reservoir of *B. crocidurae* in Senegal

While vertical transmission of TBRF *Borrelia* in ticks perpetuates endemic foci of borreliosis in the environment [98,108], small wild and peridomestic mammals constitute reservoirs [13,22,41,88,114,117,119,120]. In Senegal, more than 30 species of small rodents and eulipotyphles have been found to be naturally infected with *B. crocidurae* [38,41,59,117-121], as well as other microorganisms [30,38,45,59]. More than fourteen species of rodents, as well as a shrew and a hedgehog, have been listed as carriers of *B. crocidurae* in Senegal [37,38,54,119].

These include the rodents *Mus musculus domesticus, Mastomys erythroleucus, Mastomys natalensis, Mastomys huberti, Rattus rattus, Rattus norvegicus Arvicanthis niloticus, Cricetomys gambianus, Heliosciurus gambianus, Desmodilliscus braueri, Tatera gambiana (Gerbilliscus gambianus), Taterillus gracilis* complex, *Dasymys incomtus,* and *Myomys daltoni.* Among eulipotyphles, the species *Crocidura olivieri* and *Atelerix albiventris* are included [38,41,42,54,117-120]. Rodent populations are closely linked to vegetation cover and the nature of the soil, where most species dig burrows for shelter (Fig. 8) [59]. The Western European house mouse, *M. musculus domesticus,* a major invasive species worldwide, finds refuge in dwellings, furniture, and domestic equipment [31,38,59]. The native reddish-white multimammate mouse *M. erythroleucus,* which has multiple udders (10 pairs) and is found throughout the country but is more common in Sahelian areas, inhabits both peri-domestic dwellings and wild areas of Sudanese savannah, where it digs burrows of varying depths [43-45,59]. The African grass rat *A. niloticus* lives mainly near dwellings and in cultivated areas. It does not dig burrows but uses field fences and dense bushes to build nests and may occupy abandoned burrows [43-45,59]. The black commensal rat, *R. rattus,* was introduced to Senegal by Portuguese sailors in the 15^th^ century and spread inland via trade routes (rivers and roads). It is common in Sahelian environments, but it can also be found in Sudanian areas. The Gambian pouched rat, also known as the African giant pouched rat (C. *gambianus),* is anthropophilic and widely distributed in Sahelian and Guinean environments. The Natal multimammate mouse *(M. natalensis)* is common in Sahelian and Sudanian areas, where it lives exclusively inside dwellings. The insectivorous and carnivorous shrew *C. olivieri* is mainly found in wetlands and inside dwellings [43-45,59,115].

**Figure 8:** Distribution of *B. crocidurae* detected in different species of small mammals in Senegal

Among the main *Borrelia* reservoirs identified in Senegal (Table I, Fig. 8), a 9.2% prevalence of *Borrelia* infection was observed. This prevalence was found in both Saharo-Sahelian species, such as *Gerbillus gerbillus, G. occiduus,* and *G. tarabuli,* as well as Sudano-Sahelian species, including *A. niloticus, M. erythroleucus, and M. huberti* [42]. Small mammals captured in the Fatick region exhibited a carriage rate of 17.5% in two species of small rodents: *A. niloticus* and *M. musculus domesticus.* [88]. An infection rate of 15.2% by *B. crocidurae* was identified in five rodent species: *M. musculus domesticus, G. nigeriae, Taterillus* sp., *A. niloticus*, and *M. erythroleucus* [30]. Additionally, a prevalence of 12% was detected in invasive house mice *(M. musculus domesticus)* in the Saint-Louis region (Fig. 8) [94]. No cases of infection with *B. crocidurae* have been reported in vertebrate animal reservoirs outside the geographical range of *O. sonrai* ticks [38,117]. Data collected in Senegal, based on testing of at least 100 specimens per species, reveal that the overall prevalence of *Borrelia* infection varies by species. The prevalence was 15.3% (139/906) in *A. niloticus;* 14.7% (14/95) in *Crocidura* sp.; 11.5% (103/897) in *Mastomys* sp.; 5.7% (30/523) in *M. musculus domesticus;* and 5% (5/100) in *Gerbillus* sp. (Table I).

**Table I:** Distribution of *Borrelia infection* prevalence in small mammal species according to rainfall, climatic region (CD) and locality, Senegal

Precipitation (mm)	CD	Region	Family	Small mammals	I/T	%	Reference
50 – 250	Sahelian	Saint-Louis	Muridae	*Arvicanthis niloticus*	88/630	13,9%	38
				*Mastomys huberti*	37/235	15,7%	38
				*Mus musculus*	23/319	7,2%	38,94
				*Taterillus* sp.	1/10	10%	38
				*Gerbillus* sp.	1/20	5%	30
			Soricidae	*Crocidura* spp.	4/55	7,3%	38
250 – 500	Sahelo Sudanese	Dakar	Nesomyidae	*Cricetomys gambianus*	3/15	20%	38
			Muridae	*Arvicanthis niloticus*	4/18	22,2%	38
		Thiès	Muridae	*Mastomys erythroleucus*	24/136	17,6%	38
				*Arvicanthis niloticus*	13/94	13,8%	38
				*Gerbilliscus gambianus*	3/7	42%	38
		Louga	Muridae	*Mus musculus*	5/159	3,1%	38
				*Arvicanthis niloticus*	16/96	16,6%	30,38,72
				*Taterillus* sp.	10/53	18,8%	30,38,72
				*Gerbillus* sp.	4/80	5%	30
500 – 750	Sudanese	Fatick	Muridae	*Arvicanthis niloticus*	18/68	26,5%	38,88
				*Mastomys erythroleucus*	42/526	7,9%	38
				*Rattus* spp.	2/6	33%	38
				*Mus musculus*	2/45	4,7%	38,88
			Soricidae	*Crocidura* spp.	10/40	25%	38,88

Pluviométrie: Moyenne annuelle des précipitations de 1970 à 1999

I/T = Nombre de spécimens infectés (I)/Testés (T)

Precipitation: Average annual precipitation from 1970 to 1999

I/T = Number of specimens infected (I)/Tested (T)

## Climate and environment

The distribution of *O. sonrai* ticks in Senegal depends on climatic conditions related to rainfall frequency, temperature, soil type, and soil parameters favorable to their establishment [37,40,117]. In the western half of West Africa, the distribution of *O. sonrai* ticks appears to be independent of watercourses and other environmental factors such as vegetation, topography, mountainous areas, agricultural activity, and population density. In contrast, in the eastern part of the region, particularly in Mali, the presence of *O. sonrai* is closely linked to the river network, particularly the Niger River and its main tributaries [117]. Soils composed of fine sand mixed with coarse silt are particularly suitable for the establishment of *Ornithodoros* ticks [37,40,120]. This type of soil retains moisture, which helps maintain tick-borne borreliosis foci in the wild. Changes in soil characteristics can negatively impact the survival of ticks in their environment. Studies have shown an absence of the tick-borne borreliosis vector in certain localized areas within endemic regions, suggesting that unfavorable soil conditions may limit their presence [37,42,85].

Since 1970, climate variations in Senegal have been linked to rainfall deficits and anthropogenic action, leading to remarkable changes in ecosystems [120]. Persistent drought in the sub-Saharan region has enabled the tick *O. sonrai* to colonize new territories in the Sudanese zone, resulting in the southward expansion of the TBRF endemic area [85,104,117,120]. A study of 66 weather stations in Senegal revealed an expansion in the distribution of *O. sonrai* ticks correlated with rainfall and the southward migration of the 750 mm isohyet in the Sudanese savannah zone [37,117,120,121].

## Tick-Borne Relapsing Fever in Senegal

Most data on human *B. crocidurae* infections have been collected in Senegal (Table II). However, the CFR attributable to TBRF is not well understood [49,68,121], probably around 0.5%, but remains lower than the CFR due to *B. duttonii,* which ranges from 2 to 5% in East, South, and Central Africa [55,56,78]. Only one fatal case has been reported in the literature [55], and another case of TBRF in a pregnant woman was associated with a miscarriage in a rural area [49]. Longitudinal studies on tick-borne borreliosis have been conducted in the Dielmo-Ndiop area, located 280 km southeast of Dakar, and in Niakhar, in the Fatick region. The area’s vegetation and climate correspond to the Sudanese savannah. The population is a mixture of Wolof and Serer ethnic groups whose main activity is agriculture. Medical and epidemiological surveillance of the population in the Dielmo-Ndiop villages began in June 1990 in Dielmo and in July 1993 in Ndiop. Epidemiological and clinical data on TBRF have been reported in several research studies [36,37,39,82,88,95,121]. Patients with TBRF presented with nonspecific symptoms, including high fever (38°C to 40°C), headache, vomiting, diarrhea, back pain, arthralgia, myalgia, and abdominal pain [68,91,97]. Between 2011 and 2016, data collected at the Niakhar POC in the Fatick region showed that TBRF was the main reason for seeking care at health facilities, along with malaria and influenza [1,111]. A high prevalence of 11.7% was reported [88]. Human cases of TBRF were recorded more frequently between July and September [36,88,121]. In the Thiès region, a retrospective study of a cohort of febrile patients based on qPCR detection of plasma samples between 2018 and 2019 showed a 15% prevalence [71].

**Table II:** Prevalence and/or number of cases of tick-borne relapsing fever recorded in Senegal from 1928 to 2022

Year	Region	Area	Detection methods	Number of cases or prevalence	Reference
1928	Dakar	Town center	Thick and thin blood films	1 case of spirochecal infection	76
1932	Dakar	Town center	Thick and thin blood films	16 case of spirochecal infection	77
1934	Dakar	Town center	Thick and thin blood films	46 cases of spirochetosis	2
1942–1945	Dakar	Town center	Thick and thin blood films	198 cases of spirochetosis	10
1946	Dakar	Town center	Thick and thin blood films	85 hospitalized cases	9
1947	Dakar	Town center and Medina	Thick and thin blood films	95 hospitalized cases	13
1983	Dakar	Town center	Thick and thin blood films	23 hospitalized cases	6
1989–1990	Thiès	Keur Moussa	Thick and thin blood films	4. 2% of children aged 10–14	118
1991	Thiès	Diakhao Thialy	Thick and thin blood films	10% of borreliosis cases	36
1990–1992	Fatick	Dielmo-Ndiop	Thick and thin blood films	5,2% of borreliosis cases	119
1996	Fatick	Dielmo-Ndiop	Thick and thin blood films	10% of borreliosis cases	119
1990–2003	Fatick	Dielmo-Ndiop	Thick and thin blood films	11% of the population per year	121
2006	Fatick	Dielmo	Thick and thin blood films	11% of borreliosis cases	121
2008–2009	Fatick	Dielmo-Ndiop	Finger capillary blood PCR	11% of borreliosis cases	34
2011	Fatick	Dielmo	Finger capillary blood PCR	13% of borreliosis cases	95
2010–2011	Fatick	Niakhar	Finger capillary blood PCR	19% of borreliosis cases	82
2010–2011	Louga	Keur Momar Sarr	Finger capillary blood PCR	6% of borreliosis cases	82
2010–2011	Fatick	Dielmo-Ndiop	Finger capillary blood PCR	9,7% de cas de borréliose/ 9. 7% of borreliosis cases	82
2011–2012	Fatick	Dielmo-Ndiop	Finger capillary blood PCR	10% of borreliosis cases	111
2013–2015	Fatick	Dielmo-Ndiop	Finger capillary blood PCR	40 borreliosis cases	39
2016	Dakar	Pikine	Thick blood films and PCR	1 hospitalized case	35
2016	Fatick	Niakhar	Finger capillary blood PCR	11,7% de cas de borréliose/ 11. 7% of borreliosis cases	88
2011–2016	Fatick	Dielmo-Ndiop	Finger capillary blood PCR	4. 8% of borreliosis cases	1
2019	Fatick	Niakhar & Touba-Couta	PCR on *P. f TDR*	16% of borreliosis cases	87
2018–2019	Thiès	Thiès	Venous blood PCR	15. 5% of borreliosis cases	71
2019	Thiès	Keur Moussa	PCR on *P. f* TDR	6% of borreliosis cases	87
2019	Saint-Louis	Richard-Toll	PCR on *P. f TDR*	4% de cas de borréliose/ 4% of borreliosis cases	87
2019	Kaffrine	Kaffrine	PCR on *P. f TDR*	10% de cas de borréliose/ 10% of borreliosis cases	87
2018–2021	Diourbel	Touba	Blood PCR	5,7% des pèlerins du Grand Magal de Touba/ 5. 7% of pilgrims at the Grand Magal of Touba	57
2020–2022	Sedhiou	Bounkiling	NGS on plasma	2. 4% of borreliosis cases	93
2020–2022	Thiès	Tivaouane	NGS on plasma	6% of borreliosis cases	93
2022	8 tested regions in Senegal	Endemic regions	PCR on *P. f TDR*	216 cases of borreliosis 0. 18% to 17. 5% depending on locality	113

NGS: Next-generation sequencing

An innovative study conducted from January to December 2019 used qPCR to screen *B. crocidurae* in rapid diagnostic test (RDT) cassettes that were negative for *P. falciparum* in four regions of Senegal. The study indicated that the infection rate among non-malarial febrile patients was 7.22% [87]. The highest prevalence of *Borrelia* infection was noted in the Sudano-Sahelian zone of the Fatick (16%) and Kaffrine (10%) regions, followed by the Sahelo-Sudanese zone of the Thiès region (6%). The lowest prevalence was observed in the Sahelian zone of the Saint-Louis region (4%) [87], and cases of TBRF were observed in all seasons [1,36,87,88,111,121] (Fig. 9).

Cases of TBRF have been detected in Senegal using molecular biology techniques outside the known range of the vector tick *O. sonrai* and of small mammals naturally infected with *B. crocidurae* [26,38,80,93,117]. These cases are likely imported, as the patients had stayed in endemic areas of Senegal or West Africa. This underscores the importance of investigating travel history in patients with relapsing fever of unknown origin. Finally, cases of TBRF have been reported in travelers from endemic areas in Africa [65], with the highest number of cases identified in Senegal (Fig. 10). Between 1990 and 2021, 20 cases of imported TBRF due to *B. crocidurae* were detected, including 14 in France, 2 in Italy, 2 in Belgium, 1 in the Netherlands, and 1 in Canada (Fig. 10) [3,14,24-26,32,48,52,58,60,61,83,97,116].

**Figure 9:** Distribution of indigenous human cases of *B. crocidurae* tick-borne relapsing fever diagnosed in Senegal, 1991 -2023

**Figure 10:** Distribution of *B. crocidurae* tick-borne relapsing fever cases diagnosed in travelers returning from Senegal and neighboring countries, 1991-2021

### Diagnosis

In Senegal, as in other West African endemic countries, malaria has long been considered the primary cause of fever in the absence of clinical signs suggestive of another disease [122]. The symptoms of TBRF are non-specific and have long been confused with malaria because they are similar [116]. Cases of *Borrelia-Plasmodium falciparum* co-infections are rarely diagnosed in health facilities due to a lack of well-trained microscopists who can identify the two pathogens using thick blood smears (TBS). However, the Infectious, MINES (Maladies infectieuses, négligées et émergentes au Sud) laboratory in Dakar regularly detects *Borrelia/Plasmodium* co-infections. They were detected in febrile patients in Dielmo using the classic TBS method. These detections occurred before the advent of more sensitive molecular biology techniques, which were implemented in POCs in Niakhar and Dielmo. Due to the absence of specific clinical signs, establishing a differential diagnosis of tick-borne borreliosis is essential [19,68,91,97].

Detecting TBRF *Borrelia* is challenging in Africa due to the shortage of skilled microscopists, especially since the introduction of RDTs. Tick-borne relapsing fever (TBRF) diagnosis is based on observing spirochetes or *Borrelia* in a thick or Giemsa-stained blood smear. This technique is identical to that used to detect malaria parasites [41]. However, this technique has low sensitivity and requires a skilled technician to specifically search for *Borrelia* [41, 95]. For example, out of 27 Borrelia-positive samples detected by qPCR, only four were identified as positive by the thick smear performed in the clinic. In contrast, 15 samples were recognized as positive by well-qualified microscopists using the same technique. Intraperitoneal inoculation of blood from febrile patients or suspicious organs into white mice is a more sensitive technique than thick and blood smears [41]. However, this method is not suitable for use in healthcare facilities.

In recent years, molecular biology techniques have greatly advanced the detection of spirochetes. PCR and qPCR are the most sensitive modern diagnostic methods [95]. Our teams have implemented these techniques in small, local (POC) laboratories set up in rural areas of Senegal, first at the Dielmo-Ndiop research station [111], then in Niakhar. These POCs have enabled the detection of pathogens responsible for fevers of unknown origin and the initiation of appropriate treatments [111]. Molecular screening of these microorganisms in POCs has improved the assessment of the prevalence and incidence of TBRF in Senegal’s endemic areas [1,82,88,95,113]. Recently, MALDI-TOF MS has been used to identify *Borrelia* in cultured strains and differentiate between infected and uninfected ticks (Fig. 4) [20,50]. Finally, a new, specific diagnostic technique for rapidly detecting TBRF *Borrelia* in regions with limited resources has proven its worth [64]. These tests are called loop-mediated isothermal amplification (LAMP) and can be used in POCs set up in rural areas. The LAMP technique uses six specific primers and the necessary reagents for amplification over 60 minutes [64,92]. The high sensitivity and specificity of this test, as well as the simplicity of the DNA extraction procedure, make the LAMP technique a reliable and effective tool for diagnosing TBRF [64].

### Treatment

Like other agents responsible for TBRF, *B. crocidurae* is sensitive to many antibiotics readily available at pharmacies. The preferred antibiotic treatment for TBRF is doxycycline. For adults, the recommended dosage is 100 mg twice daily. For children, the dose is 4 mg/kg in a single dose [68]. Erythromycin has been used to treat pregnant women and children under eight years of age [16,68]. There is limited evidence regarding the efficacy of azithromycin or amoxicillin. For severe or neurological forms of TBRF, or in cases of vomiting, a treatment option is intravenous and/or intramuscular ceftriaxone, depending on the stage of the disease [12,67]. *Borrelia* are resistant to rifampicin, metronidazole, and sulfonamides [68].

## Control of tick-borne relapsing fever in Senegal

Traditional methods of controlling vector-borne diseases often rely on chemical insecticides and/or acaricides for spraying or dusting to reduce or control vectors. These methods pose contamination risks to rural populations who are often poorly informed about the associated risks. Added to this are the specific ecological constraints of *Ornithodoros* ticks, which are ticks that live inside burrows (endophiles), and resistance issues. For this reason, a non-chemical alternative strategy has been developed to control tick-borne borreliosis in endemic areas of Senegal.

From 2013 to 2015, it was implemented in Dielmo and Ndiop [39]. In rural areas of Africa, traditional houses are made of rammed earth with unpaved floors, sometimes paved but often in poor condition [39]. The goal of this strategy was to cement the floors of bedrooms, attics, and kitchens. The main objective was to eliminate small rodent burrows to reduce the risk of contact with tick vectors [39]. The strategy targeted three main actions. First, it aimed to eliminate human-tick contact by filling all burrows and crevices in the ground with a cement and clay mixture about 5 cm thick. Second, it involved regularly and systematically monitoring the appearance of new burrows and crevices in order to quickly close them [39]. Third, small mammals were captured using non-toxic liquid glue to reduce their presence in homes and thus limit the spread of ticks. At the same time, awareness of sanitary hygiene was raised by regularly sweeping the inside of dwellings to eliminate stray ticks outside burrows and reduce the risk of human-tick contact [39]. These measures, implemented with collective involvement from the population, led to a significant reduction in relapsing tick fever incidence. In Dielmo, for instance, the incidence rate decreased by 89.8%, dropping from 10.55 to 2.63 cases per 100 person-years. In Ndiop, the decline was 81.5%, from 3.79 to 1.39 cases per 100 person-years [39]. A total of 36 borreliosis infections were prevented at an economic cost of €526 (345,033 CFA francs) per infection prevented. Since January 2016, this strategy and its accompanying measures have been transferred to the community level [39]. Equipment, such as liquid glue and cement, continues to be provided to capture small mammals and seal burrows in homes. In 2017, this provision was discontinued, but the population permanently adopted the preventive control strategy [39]. Today, human cases of borreliosis are rare or non-existent in these two villages.

## Conclusion

In the context of malaria pre-elimination, TBRF is the primary reason for seeking medical attention for fever-related symptoms in many health facilities in rural, endemic areas of Senegal. Studies conducted in Senegal over the past 30 years on tick-borne borreliosis show that this disease is a significant health concern in rural areas. However, TBRF is not well known among clinicians and authorities. It is rarely suspected or diagnosed as the cause of fever, except in pilot centers where POC laboratories have been set up. This review is intended for decision-makers and health authorities, both national and international. It emphasizes the need for greater awareness of this neglected public health problem. The northern two-thirds of the country, comprising ten regions (Dakar, Thiès, Diourbel, Louga, Saint-Louis, Matam, Fatick, Kaolack, Kaffrine, and Tambacounda), are endemic, characterized by a massive presence of *O. sonrai* ticks infected with *B. crocidurae* in the wild and in human dwellings. Additionally, small mammals that carry the infection are constantly present in houses, favoring the persistence of the disease. This situation underscores the importance of including tick-borne relapsing fever (TBRF) caused by *B. crocidurae* in the list of neglected tropical diseases (NTDs). It is also crucial to establish an epidemiological surveillance program for this disease. Finally, mass awareness campaigns must be organized among populations living in endemic regions to better prevent and control borreliosis (Fig. 9).

The preventive control strategy implemented in Dielmo and Ndiop, along with supporting measures, has significantly reduced the incidence of the disease. This success is due to the involvement and commitment of the local populations. This approach should be scaled up in all endemic rural areas of Senegal and West Africa. It could be incorporated into a national or sub-regional program aimed at minimizing economic losses related to absenteeism and the improper treatment of non-malaria fevers.

To better manage TBRF cases rapidly and effectively in Senegal, POCs should be established as a priority in endemic regions, followed by other regions. This would enable more precise monitoring and management of TBRF cases, especially among patients presenting with non-malaria fever. Additionally, there is ongoing advanced thinking on the development of a rapid diagnostic test (RDT) specific to *B. crocidurae.* This easy-to-use, accessible device would facilitate rapid diagnosis and improve the management of TBRF cases in Senegal’s main endemic areas, as well as in other West and North African countries.

## Acknowledgments

The authors would like to thank Jean-François Trape and Didier Raoult for their invaluable assistance in locating information and publications, including older scientific articles that are not available online. We are also grateful for their contributions to our understanding of the epidemiology of TBRF in Senegal, as well as for all of their work on this disease.

## Funding

This study was supported by the Institut Hospitalo Universitaire (IHU) Méditerranée Infection; the Agence Nationale de la Recherche (ANR), as part of the “Investissements d’Avenir” program (reference ANR-10-IAHU-03); and the Alpes-Côte d’Azur Region.

## Institutional review board statement

The Senegalese National Ethics Committee for Health Research (CNERS) approved this study under protocol number 00081MSAS/DGS/DS/CNERS in 2012.

## Ethical statement

The authors declare that all data were collected, processed, and analyzed in accordance with ethical regulations and applicable laws on personal data protection. All information that could identify study participants has been anonymized to ensure their confidentiality and privacy, in accordance with institutional guidelines and approved protocols for processing sensitive data. The sources of the data have been properly cited in the manuscript.

## Authors’ Contributions

NDIAYE El Hadji Ibrahima: Data collection, methodology, and writing.

DIATTA Georges: Methodology and data validation.

SOKHNA Cheikh: Conceptualization, methodology, and data validation.

PAROLA Philippe: conceptualization, methodology, data validation, and supervision.

## Conflicts of interest

The authors declare that they have no conflicts of interest.
